# Real-time characterisation of microbe-induced inflammation using a novel zebrafish larval corneal injury and infection model

**DOI:** 10.1038/s42003-026-09985-1

**Published:** 2026-04-14

**Authors:** Kelvin K. W. Cheng, Carl S. Tucker, Justyna Cholewa-Waclaw, Stephen Mitchell, Fraser Laidlaw, Bethany Mills, Adriano G. Rossi

**Affiliations:** 1 Centre for Inflammation Research, Institute for Regeneration and Repair, University of Edinburgh, Edinburgh, UK; 2 Bioresearch & Veterinary Services (BVS) Aquatics Facility, College of Medicine and Veterinary Medicine, University of Edinburgh, Edinburgh, UK; 3 High Content Screening Facility, University of Edinburgh, Edinburgh, UK; 4 Kings Buildings, University of Edinburgh, Edinburgh, UK

**Keywords:** Imaging the immune system, Innate immunity, Translational research

## Abstract

Microbial keratitis (MK) is a major global cause of blindness. Yet, treatment is heavily dependent on antimicrobials with limited options for immunomodulators - despite the critical role of dysregulated immune responses in disease pathogenesis. This gap reflects a critical unmet clinical need and is compounded by the lack of model systems capable of real-time high-resolution immune dynamics analysis. To address this, we developed a zebrafish larvae MK model utilising transgenic zebrafish lines with fluorescently labelled neutrophils, macrophages and basal epithelial cells. Corneal injury triggered rapid immune cell recruitment which was amplified by exposure to pro-inflammatory mediators such as N-formylmethionine-leucyl-phenylalanine (fMLF) and leukotriene B_4_ (LTB_4_). Infection with live bacteria induced robust, sustained neutrophil and macrophage recruitment, marked by increased neutrophil speed and migratory distance. This model enables dynamic in vivo visualization of immune cell dynamics, offering a powerful and scalable platform to accelerate the discovery and screening of novel immunomodulators for MK.

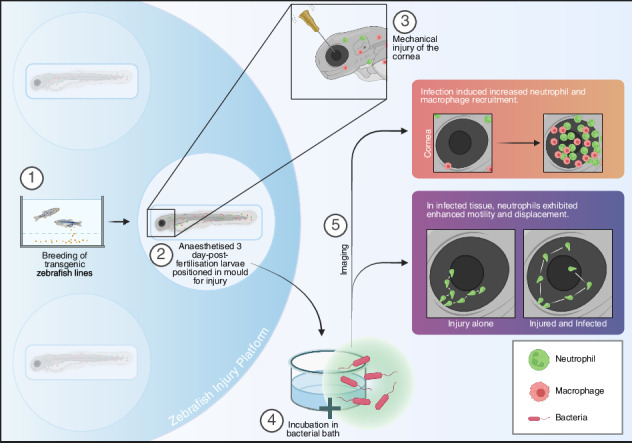

## Abstract (web summary)

A zebrafish larvae model of microbial keratitis enables real-time analysis of immune cell migration following corneal injury and infection and reveals increased neutrophil motility during *Pseudomonas aeruginosa* infection

## Introduction

MK infection of the cornea is the commonest cause of non-trachomatous corneal opacity globally, and is the fifth most common cause of blindness worldwide, accounting for 3.5% (36 million) of all blind patients from 1980 to 2014^1^. Described as a ‘silent epidemic’, MK remains a major global health problem, particularly affecting the low- and middle-income countries, with patients suffering from pain, vision loss and prolonged disease^2^. The need for improving MK treatment was highlighted as the leading research question in corneal research by the UK Clinical Eye Research Strategy Priority Setting Partnership in 2024 for the next 5 years^3^. At present the treatment strategy for MK has largely been dependent on eradication of pathogens with antimicrobials. Antimicrobials alone do not address the dysregulated inflammatory response responsible for much of the tissue damage morbidity and mortality observed occurring in inflammatory diseases. Specifically the well-recognised detrimental effect of excessive inflammation in MK can lead to corneal melting and fibrosis^4^, compromising the patient’s vision in the long-term with few non-invasive therapeutic options. Elucidating the mechanistic basis of the immune response to MK will therefore enable the discovery of innovative immunomodulatory therapies.

An ideal animal model for studying the immune response should possess anatomical similarity and genetic homology to humans be cost-effective, offer high genetic tractability and allow for in vivo dynamic intravital imaging to monitor the behavioural patterns of immune cells. These features collectively are absent in any of the existing animal MK models^5^ which typically rely on mouse or rabbit models to study MK.

Zebrafish have proven a useful model organism to study multiple areas of biology^6^ including inflammatory diseases^7–10^, cancer^11^, rare genetic diseases^12^ and cardiovascular diseases^13^. The optical clarity of zebrafish larvae allows for dynamic in vivo tracking of multiple types of fluorescently labelled cells. This provides an unmatched ability to study behaviours of the innate immune response to injury or infection over a protracted period in the same fish reducing the number of protected animals required to achieve the same endpoint with traditional mouse models, supporting the NC3R principles of replacement, reduction and refinement^14^.

Extensive insights into immune cell behaviour have been obtained using the zebrafish model system^15–17^ both in inflammation and infection biology^18,19^. For instance, studying *Mycobacterium marinum*, which induces a tuberculosis-like disease in zebrafish, has significantly advanced our understanding of *Mycobacterium tuberculosis infection* in humans^20^. The tail amputation model^21^ has proved invaluable for understanding immune response signalling pathways^18^ and identification of potential drug candidates^22^. Notably this system provided the first in vivo evidence of reverse migration of neutrophils as a mechanism of clearing these cells from the interstitium of wounded tissue^23^. Furthermore the use of larvae during the first two weeks post-fertilisation allows focused interrogation of the innate immune system, since the adaptive immune system only becomes functional after 3–6 weeks of development^24^. Having platforms that enable the interrogation of host–pathogen interactions is crucial for deeper understanding of the immune response mechanics. The zebrafish cornea shares strong conservation of features both structurally having similar layered organisation and possession of a distinct peripheral limbal stem cell niche like humans, and molecularly, with several molecular markers such as keratan sulfate and keratin 3 being expressed in both species^25^.

Here we report a novel zebrafish larval model of MK. Such a model offers a new perspective on MK pathogenesis by allowing the dissection of host–pathogen interactions in real-time and manipulation of the immune response through pharmacological or genetic means via use of morpholinos^26^ (synthetic antisense oligonucleotides that modify specific gene expression) and clustered regularly interspaced short palindromic repeats-Cas9 (CRISPR-Cas9) approaches^8^—capabilities not easily achieved in current in vivo models. Additionally, when utilised at scale, this model holds the potential for high-throughput drug screening of novel therapeutics for MK. We validate the model by describing the baseline immune response to injury and perturb the immune response using human pro-inflammatory mediators and live bacteria. By enabling detailed observation of individual immune cell behaviours (such as motility and intercellular interactions), this model facilitates greater understanding of the immune response to both injury and infection. This approach paves the way for identifying targeted immunomodulators to precisely influence cell behaviour at various stages of MK.

## Results

### Development of a zebrafish larvae corneal injury model using the zebrafish injury platform (ZIP)

**Fig. 1:**
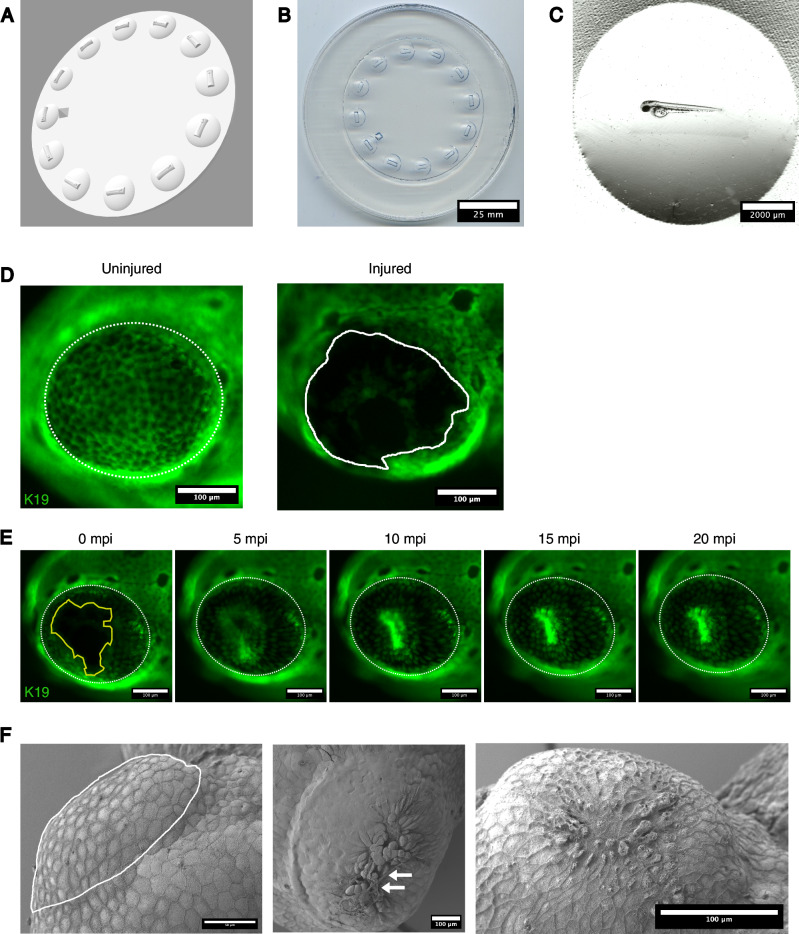
The zebrafish injury platform (ZIP) and validation of corneal injury technique. **A** 3D modelling of the zebrafish injury platform (ZIP) cast developed using Tinkercad. **B** The resulting mould formed using 2% agarose to house zebrafish larvae while performing injury. Scale bar = 25 mm. **C** Exemplar image of a 3-day-post-fertilisation (dpf) zebrafish larvae within the mould. Scale bar = 2000 µm. **D** Representative images of the corneal epithelium before injury (white dotted line) and the wound after mechanical injury (white solid line) of a *Tg(K19:GFP)* zebrafish larvae, with green fluorescent basal epithelial cells. Scale bar = 100 µm. **E** Representative time course images of the zebrafish larvae cornea (white dotted line) following injury (yellow solid line) in a *Tg(K19:GFP)* zebrafish larvae. Minutes post-injury (mpi). Scale bar = 100 µm. **F** Exemplar scanning electron microscopy images of zebrafish larvae cornea before injury (left) (white solid line) 15 min post-injury (middle), and 4 h post-injury (right). The white arrows indicate web-like structures in the closing wound which could be fibrin or extracellular traps or a combination of both. Scale bar = 50 µm left image, 100 µm middle and right images.

Mechanical injury to the cornea is the leading risk factor for establishing MK^27^ and therefore was chosen as the method of inducing injury in the model. To perform reproducible damage to the cornea across our zebrafish population, we developed the zebrafish injury platform (ZIP). ZIP is a bespoke mould designed to follow the contours of the body of zebrafish larvae between 72- and 120-h post-fertilisation (hpf), compensating for the larvae’s irregular body shape (Fig. 1A–C), thereby ensuring consistent horizontal and lateral positioning and effective counter-traction. The ZIP design also reduced dehydration-related deaths and fin damage by using individual water reserves to keep each larva hydrated until just before injury, unlike other flat surfaces (such as glass or agar).

The ZIP was highly effective for enhancing the efficiency and consistency of corneal injury allowing for many larvae to be processed per experimental round (24 zebrafish larvae per h). Each cornea was injured with ten linear scratches limbus-to-limbus, covering the entire corneal surface using a 30G needle dipped in 20% ethanol. This technique resulted in detached sheets of epithelial cells a positive indicator that the injury had been executed successfully. On average 59.6% (*n* = 34) of the corneal surface was debrided with exposure of corneal stroma, confirmed by fluorescence microscopy of the *Tg(K19:GFP)* transgenic zebrafish line, which has fluorescent basal epithelial cells (Fig. 1D) and scanning electron microscopy (SEM) (Supplementary Fig. 1A).

Immediately after injury the surrounding epithelial cells rapidly extended and migrated centripetally, closing the wound within 15 min (Fig. 1E and Supplementary movie 1). The migrated cells created a central ridge of clustered basal epithelial cells (Supplementary movie 2) which gradually smoothed out over 24 h. In several samples, web-like structures were also observed on the wound surface (Fig. 1F middle tile, and Supplementary Fig. 1C, D). This could represent the formation of fibrin clots, neutrophil extracellular traps (NETs)^9,28^ or a combination of both which requires further investigation. The NETs seen rapidly post-injury may represent vital NETs which have been reported to form rapidly, within 5–60 min post-stimulation^29^, by mediators such as C5a or TLR4 stimulation on platelets^30^. We hypothesise that fibrin clots form rapidly and this could then stimulate the formation of vital NETs by recruited neutrophils. Nonetheless these findings are preliminary and will require further characterisation. Additionally studying post-injury tissue healing wound mechanics and immune resolution could be further deepened using the *Smad3/ TGF-β*reporter line^31^ as TGF-β is important in mediating wound healing in most tissues, including the cornea^32–35^.

While we found that the wound had closed by 15 min post-injury surface irregularities were still present at 4 hpi (hours post-injury), as determined by SEM (Fig. 1F, far right).

### Zebrafish corneal injury triggers immediate neutrophil influx and subsequent macrophage response

Neutrophils and macrophages are key cells of the innate immune response and are early responders to sites of injury and infection. Although neutrophils play a pivotal role in pathogen clearance, persistence of these cells is associated with tissue injury due to collateral inflammation^36,37^. While it is known that neutrophils accumulate within the cornea within the first 2 h of injury^38^, little is known about their precise role in early MK, interactions with other cell types such as macrophages, and their responses to specific mediators remain largely unclear. A greater understanding of immune cell dynamics within the cornea is crucial given its distinct tissue architecture and characteristics, including its avascular^39^ and immune-privileged nature^40^.

**Fig. 2:**
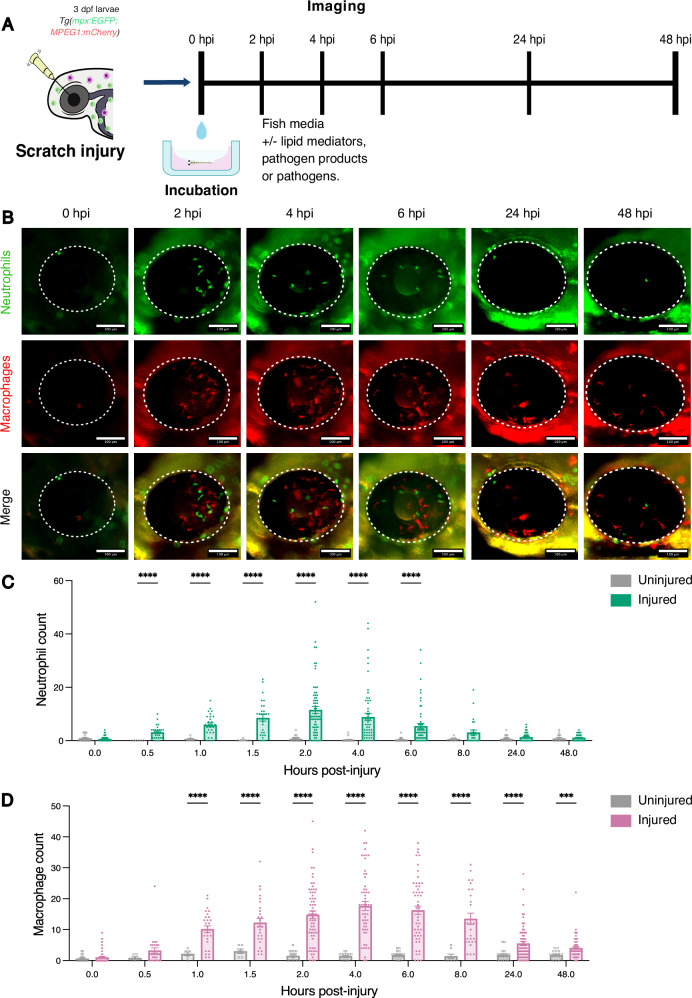
Baseline characterisation of neutrophil and macrophage migration over 48 h using the zebrafish larvae corneal injury model. **A** Schematic of the zebrafish larvae corneal injury model. **B** Representative images of injured zebrafish larvae corneas of the transgenic line, *Tg(mpx: EGFP; MPEG1:mCherry)* depicting neutrophil (green) and macrophage (red) migration into the cornea. Scale bar = 250 µm. **C** Quantification of neutrophil migration into the cornea post-injury (filled symbols) and in uninjured larvae (unfilled symbols), based on epifluorescence imaging. **D** Quantification of macrophage migration into the cornea post-injury (filled symbols) and in uninjured larvae (unfilled symbols), based on epifluorescence imaging. *n* = 66 in the injured group and *n* = 20 in the uninjured group across six independent experiments. Two-way ANOVA with post-hoc analysis using Bonferroni’s multiple comparison test and unpaired t-tests. Error bars indicate mean ± SEM. *P*-values: *P ≤ 0.05; **P ≤ 0.01 and ***P ≤ 0.001.

To measure neutrophil and macrophage responses to corneal injury we utilised a zebrafish double transgenic line *Tg(mpx: EGFP; MPEG1:mCherry)*, with fluorescently labelled neutrophils and macrophages. Using this line examination of immune cell migration in response to injury was quantified. Corneal injury resulted in a significant influx of neutrophils and macrophages into the zebrafish cornea with a peak in neutrophil and macrophage accumulation at 2 hpi and 4 hpi, respectively (Fig. 2A, B). While we observed a rapid increase of both neutrophils and macrophages following injury, there were significantly more macrophages than neutrophils within the cornea from 4 hpi onwards (*P* < 0.001). Neutrophil numbers decreased rapidly after 2 hpi reaching baseline levels within 6–8 h while macrophages persisted for longer, only reaching baseline levels at 24 h. Notably the extent of immune cell migration (immune response) was not associated with the size of the corneal injury (Supplementary Fig. 2A, B).

To enable comprehensive analysis of cellular interactions during corneal inflammation which we will describe later, we required simultaneous visualisation of basal epithelial cells, neutrophils and macrophages. This necessitated the development of an alternative transgenic line with compatible fluorescent reporters. Since the *Tg(K19:GFP)* line labels basal epithelial cells with GFP we could not use our standard *Tg(mpx:EGFP)* neutrophil reporter due to spectral overlap. To address this technical constraint, we generated the triple-transgenic line *Tg(K19:GFP;LysC:mTurquoise;mfap4:tdTomato-CAAX)*, which employs LysC-driven mTurquoise for neutrophils^41^ and mfap4-driven tdTomato-CAAX for macrophages^42^. This fluorophore combination (GFP/mTurquoise/tdTomato) provided distinct spectral separation allowing us to simultaneously track epithelial, neutrophil and macrophage populations in the experiments. Here we validate that this line with alternative promoters for neutrophils and macrophages show similar patterns of immune cell migration (Supplementary Fig. 2C).

### Neutrophil and macrophage infiltration in response to corneal injury is perturbed by exogenous pro-inflammatory mediators

**Fig. 3:**
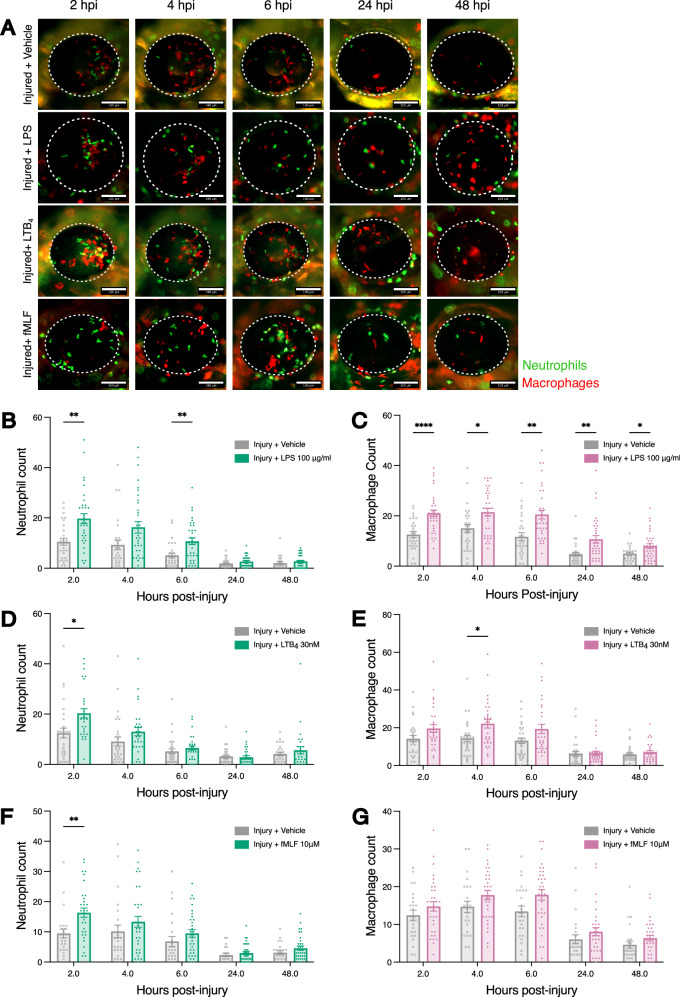
A transient raised inflammatory response is observed with the addition of well-established pro-inflammatory mediators. **A** Representative images of injured zebrafish *Tg(mpx:EGFP; MPEG1:mCherry)* larvae corneas (white dashed lines) with the addition of vehicle or mediators (LPS, LTB_4_ and fMLF). Neutrophils fluorescence in green and macrophages in red. **B** Neutrophil and **C** macrophage counts with the addition of 100 µg/L LPS (*n* = 34 in treated and 27 in vehicle group). **D** neutrophil and **E** macrophage counts with the addition of 30 nM LTB_4_ (*n* = 30 in both groups). **F** neutrophil and **G** macrophage counts with the addition of 10 µM fMLF (*n* = 33 in treated and 24 in vehicle group). Results obtained over a minimum of 3 separate experiments. Scale, 100 µm. Two-way ANOVA with post-hoc analysis using Bonferroni’s multiple comparison test and unpaired t-tests. Error bars denote mean ± SEM. *P*-values: *P ≤ 0.05; **P ≤ 0.01 and ***P ≤ 0.001.

While the immune cell recruitment was independent of injury area in our sterile inflammation model, it was significantly increased by the addition of pro-inflammatory mediators, such as those derived from the cell-envelope of gram-negative bacteria, lipopolysaccharide (LPS). A 2-fold increase in both neutrophil (2 hpi, *P* = < 0.01) and macrophage (4 hpi, *P* = < 0.05) number was measured in the injured cornea of LPS-treated larvae, compared to larvae with sterile injured corneas (Fig. 3A–C).

This heightened immune response to microbial components highlights the potential of using this model to explore therapeutic strategies aimed at modulating inflammation. Indeed a key approach gaining traction for a range of corneal disorders, including dry eye, peripheral ulcerative keratitis and MK is through the modulation of the immune response^43–45^. As proof-of-concept we demonstrated that well-established chemoattractants (LTB_4_ and fMLF, see below) can manipulate immune cell migration in zebrafish cornea, akin to their human counterparts, hence confirming that our zebrafish model is a useful complementary model for studying immune responses to human corneal diseases.

Leukotriene B_4_ (LTB_4_) is a chemoattractant for a range of leucocytes including neutrophils and macrophages^46^. When LTB_4_ was added to the fish media post-injury the number of neutrophils and macrophages within the cornea at the 2–4 h peak increased significantly by 1.6-fold (*P* = 0.0261) and 1.5-fold (*P* = 0.0388), respectively, compared to larvae with sterile-injured corneas (Fig. 3A, D, E). Similarly, N-formylmethionine-leucyl-phenylalanine (fMLF), another leucocyte chemoattractant resulted in a significantly higher neutrophil count at its peak (1.7-fold, *P* = 0.0094), compared to sterile-injured corneas. However, while macrophage numbers in fMLF-treated larvae were higher than sterile-injured corneas, the increase (1.3-fold) did not achieve statistical significance (Fig. 3A, F, G).

### A persistent, sustained inflammatory response is observed with the addition of bacteria

**Fig. 4:**
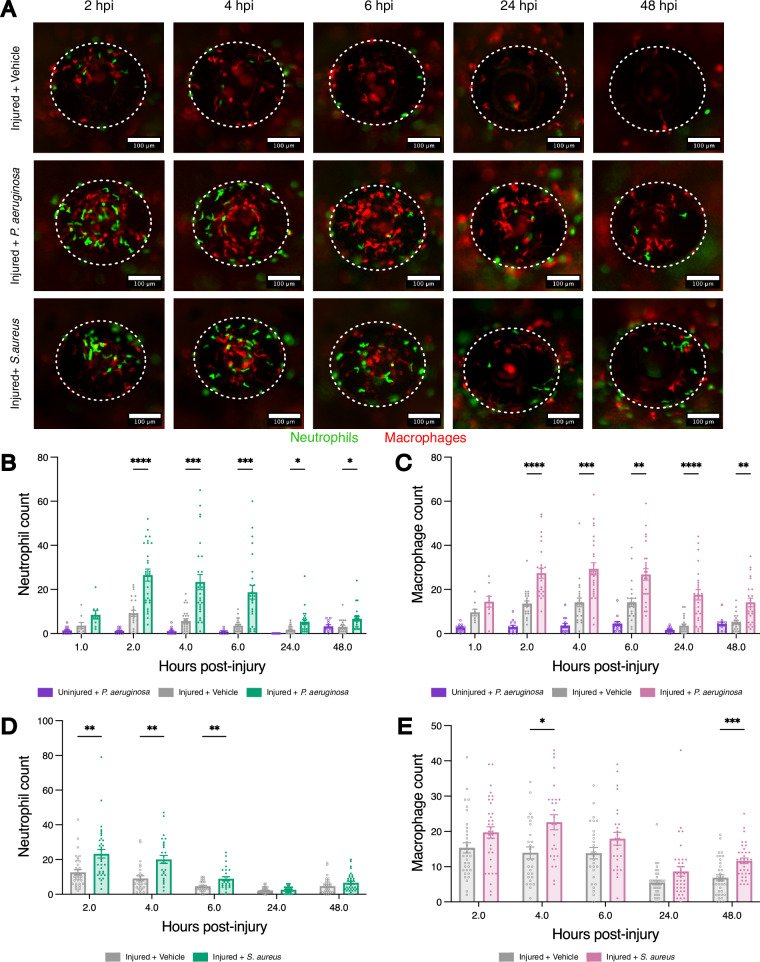
A persistently raised inflammatory response is observed with the addition of live bacteria. **A** Representative images of injured zebrafish *Tg(mpx:EGFP; MPEG1:mCherry)* larvae corneas (white dashed lines) with the addition of vehicle or live *Pseudomonas aeruginosa* or *Staphylococcus aureus*. Neutrophils fluorescence in green and macrophages in red. **B** Quantification of neutrophil counts with the addition of *P. aeruginosa* from epifluorescence images (*n* = 27 in infected group and 24 in vehicle group). **C** Quantification of macrophage counts with the addition of *P. aeruginosa* from epifluorescence images (*n* = 27 in infected group and 24 in vehicle group). **D** Quantification of neutrophil counts with the addition of *S. aureus* from epifluorescence images (*n* = 36 in both groups). **E** Quantification of macrophage counts with the addition of *S. aureus* from epifluorescence images (*n* = 36 in both groups). Results obtained over a minimum of 3 separate experiments. Scale, 100 µm. Two-way ANOVA with post-hoc analysis using Bonferroni’s multiple comparison test and unpaired t-tests. Error bars denote mean ± SEM. *P*-values: *P ≤ 0.05; **P ≤ 0.01 and ***P ≤ 0.001.

To investigate the immune response in the context of corneal infection injured zebrafish were incubated in a bath containing common ocular pathogens; *Pseudomonas aeruginosa* (gram-negative bacteria) (Supplementary Fig. 1B), and *Staphylococcus aureus* (gram-positive bacteria) (Fig. 4A). Importantly exposure of uninjured larvae to a bacterial bath did not trigger immune cell migration to the cornea (Fig. 4B, C); a significant immune cell influx was only observed when the cornea was injured -mirroring the response seen in human MK.

Compared to larvae with injury alone those exposed to *P. aeruginosa* (Fig. 4A–C) showed an approximately 3-fold and 2-fold increase in the neutrophil and macrophage number, respectively at their peak (neutrophils *P* < 0.0001; macrophages *P* = 0.0002). The number of immune cells recruited was consistently higher compared to injury alone throughout the 48 h period. Similarly the neutrophil and macrophage cell number increased 2- (*P* = 0.0023) and 1.3-fold (*P* = 0.0124), respectively, at their peak in response to *S. aureus* following injury (Fig. 4A, D, E). While neutrophil numbers decreased to baseline levels at 48 hpi macrophage numbers remained significantly elevated (1.7-fold, *P* = 0.0005).

### Increased neutrophil accumulation secondary of *P. aeruginosa* infection is associated with augmented neutrophil speed

**Fig. 5:**
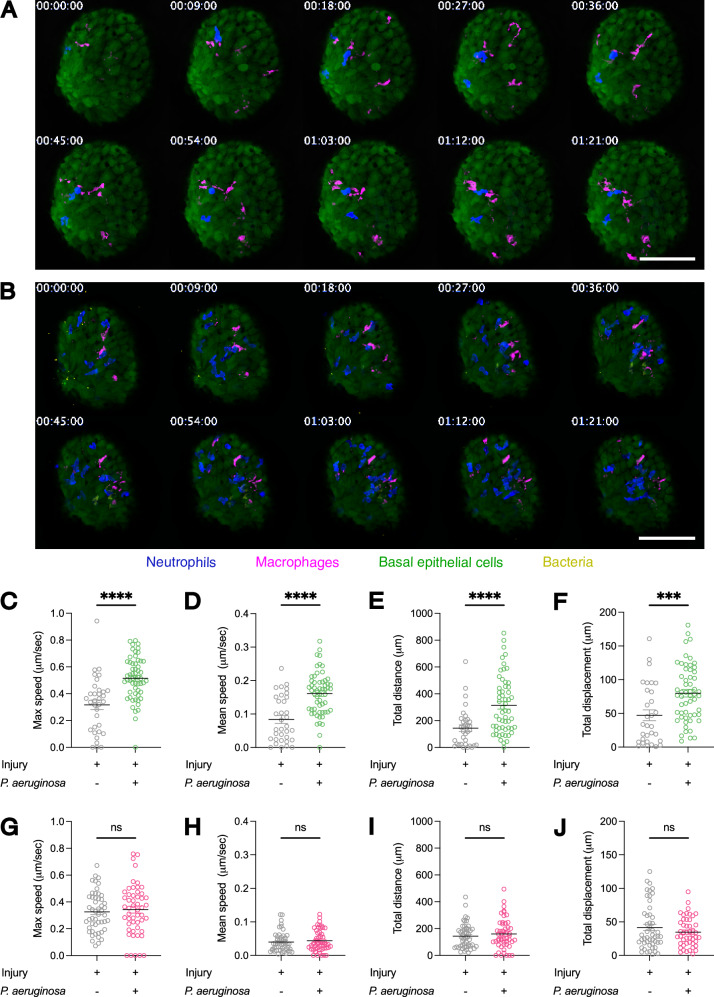
Dynamic changes in neutrophil motility observed with *P. aeruginosa* infection. **A** Montage of images of a sterile injured cornea compiled as a maximum projection 1 h post-injury. The *Tg(K19:GFP; LysC:mTurquoise; mfap4:tdTomato-CAAX)* transgenic zebrafish line was utilised for the experiment. Scale bar = 100 µm. Neutrophils shown as blue and macrophages as pink. **B** Montage of images of injured cornea compiled as a maximum projection 1 h post-injury, infected with *P. aeruginosa*. Scale bar = 100 µm. Neutrophils shown as blue, macrophages as pink and bacteria as yellow. Quantification of neutrophil **C** maximum and **D** mean speed, **E** total distance and **F** displacement (*n* = 31 in sterile injury group and 56 in injured and infected group). Quantification of macrophage **G** maximum and **H** mean speed, **I** total distance and **J** displacement (*n* = 50 in sterile injury group and 52 in injured and infected group). Cells observed in 5 injured and 6 injured and infected larvae from 4 separate experiments. Unpaired t-tests. Error bars denote mean ± SEM. *P*-values: *P ≤ 0.05; **P ≤ 0.01; ***P ≤ 0.001 and ****P ≤ 0.0001.

To understand the factors contributing to increased accumulation of neutrophils within the injured and infected cornea (Fig. 5A, B) we sought to study the motility of recruited neutrophils and macrophages individually within the injured corneas. To account for the high neutrophil speed, a spinning disc confocal microscope was utilised to image the infected zebrafish corneas at 20 s intervals.

Consistent with our observations using widefield microscopy the wound closed rapidly, and this was closely followed by the accumulation of neutrophils. Both neutrophils and macrophages migrated from the peripheral cornea with neutrophils accumulating at a faster rate than macrophages. At 1 h post-sterile injury the mean speed of neutrophils was approximately double that of macrophages (0.8 and 0.4 µm/s respectively).

Phagocytosed fluorescently labelled bacteria by neutrophils and macrophages validate that the immune response observed is not only due to mediators released by bacteria within the bath but due to direct invasion of the bacteria within the wounded tissue (Supplementary movie 3). The maximum and mean neutrophil speed increased by 1.6- and 1.9-fold, respectively with the addition of bacteria (both *P* < 0.0001; Fig. 5C, D and Supplementary movie 4). This also reflected in a greater total distance (2.2-fold, *P* < 0.0001) and displacement (1.7-fold, *P* < 0.001) of neutrophils (Fig. 5E, F). In contrast macrophage speed and distance travelled were similar in both sterile injury and infected corneas (Fig. 5G–J) over the period studied. There was no difference in the meandering index, mean directional change rate or duration within the cornea of both neutrophils and macrophages in sterile and infected corneas (Supplementary Fig. 3A–F).

## Discussion

Here we report the development and characterisation of a novel zebrafish larvae model of corneal injury and infection (MK), enabling detailed study of the immune response while leveraging the benefits of the zebrafish system. Using transgenic zebrafish with fluorescently labelled immune and epithelial cells, the innate immune response can be interrogated dynamically over an extended period without the need for culling them for further processing. Given their high reproductive rate and small size, zebrafish allow for larger sample sizes, thereby enhancing the statistical power of experiments. The zebrafish corneal anatomy has been described in detail highlighting the many similarities between corneas of both species^25,47^. Notably, corneas from both species contain the Bowman’s layer, thought to play an important role in corneal wound healing. The presence of this layer in murine corneas is controversial. The unequivocal presence of the Bowman’s layer in zebrafish corneas therefore offers an additional advantage over murine models. To enable us to take advantage of larger sample sizes for our in vivo study we found it necessary to develop the ZIP. The bespoke mould facilitated the process of inducing injuries to the cornea improving both accuracy and consistency. While there are moulds or devices publicly available to position zebrafish larvae for experimentation, none of them^48–53^ allow effective scratching of the zebrafish eye in the high numbers required for this model. To facilitate adoption of the model, the ZIP 3D printing design file is available from Figshare^54^. Working with the zebrafish ocular anatomy, we were able to utilise the dark iris layer as a unique landmark to signify the depth of imaging required to count inflammatory cells recruited into the cornea.

We established that the mechanical injury induced a robust inflammatory response that could be accurately tracked over time within the cornea. With exposure to classic pro-inflammatory mediators such as LTB_4_ and fMLF, a heightened inflammatory response was observed, in line with previous observations in mammalian and zebrafish models^8,55,56^. These inflammatory mediators were selected for their established relevance to bacterial keratitis. Formyl peptides such as fMLF act as pathogen-associated molecular patterns (PAMPS) released during bacterial infection and drive neutrophil chemotaxis via formyl peptide receptors^57,58^. LTB_4_ is a potent lipid chemoattractant produced by corneal epithelial cells in response to bacterial stimuli^59^ is elevated in human tear fluid during ocular surface inflammation, including bacterial keratitis and contact lens-associated disease^60,61^ and correlated with neutrophil recruitment in mouse models of *P. aeruginosa* keratitis. Employing these mediators enables mechanistic dissection of immune-cell recruitment downstream of clinically relevant inflammatory signals while maintaining experimental control and reproducibility.

We hypothesise that the relatively short-lived response induced by the pro-inflammatory mediators was due to the rapid wound closure; thereby restricting further exposure of immune cells to the mediators. This is supported by further amplification of the immune cell recruitment in response to live bacterial insult following corneal injury. It is widely known that bacterial infections increase neutrophil recruitment^62,63^, and we have demonstrated this here. Further by taking advantage of the unique cornea anatomy (being a thin avascular planar tissue) and high-speed confocal microscopy, we show for the first time that an increase in neutrophil speed potentially contributes to the increased neutrophil recruitment in MK. Our observation that neutrophils which exhibited fewer branches, migrated faster than macrophages support the principle that a trade-off exists between cell polarity and branching: highly branched cells explore more directions but move less persistently^64^. Future studies using this model could investigate how cell migration during dynamic wound healing influences decision-making and the regulation of cell polarity.

While our approach which imaged immune dynamics only during the early post-injury window, does not allow definitive conclusions regarding neutrophil fate during inflammation resolution (e.g. reverse migration, apoptosis or dispersal), it nevertheless captures the acute inflammatory response. Future studies employing photoconvertible reporters (such as Dendra2) and longer-term imaging will be well-suited to address these questions and further refine the mechanisms of inflammatory resolution in the cornea^65^.

The immune-cell kinetics observed in our zebrafish larvae model closely recapitulate the fundamental inflammatory trajectory of mammalian systems albeit on an accelerated timescale. In mouse models, neutrophils act as the primary responders, with infiltration occurring as early as 2 h post-abrasion^38^ or 6 h following stromal LPS injection^66^. We observe a similarly rapid primary neutrophil influx in the zebrafish followed by a secondary wave of macrophages. While mammalian macrophage populations typically peak much later and continue to rise for up to 7 days post-insult^67^ the zebrafish model demonstrates a compressed version of this hierarchical sequence. This conservation of temporal hierarchy underscores the translational utility of the zebrafish for modelling MK.

We hypothesise that neutrophil speed is altered in response to several factors including greater chemokine gradients in infected tissues and types of chemokines released. Infections are typically characterised by the presence of PAMPS while sterile injuries primarily involve the release of damage-associated molecular patterns. It is well established that neutrophils prioritise pathogen-derived factors such as complement factor 5a and formylated peptides compared to host-secreted-factors such as interleukin-8 and LTB_4_^68^. This is in agreement with Richardson et al., who showed that in vitro neutrophil speed and ROS production were differentially increased in response to specific strains of bacteria^69^. In addition specific strains of bacteria have been demonstrated to elicit different neutrophil responses with *Streptococcal* strains being potent inducers of heparin-binding protein and resistin, both reportedly associated with sepsis severity^70^.

Macrophage speed remained unchanged in infected tissues at this early stage of infection speculated to reflect their key roles in clearing cellular debris and promoting tissue repair later in the infection course.

We have characterised our cornea injury/infection model over the non-protected stages of zebrafish life and therefore our observations are most reflective of the initial stages of MK pathogenesis. The inflammatory response described here predominantly reflect the innae immune response given the absence of a fully developed adaptive immune system in the zebrafish larvae. While this restricts direct modelling of later stages of MK—where adaptive immunity contributes to disease progression—it also represents a strength of the system by enabling high-resolution analysis of innate immune responses in isolation. Given the critical role of innate immunity in initiating MK, this approach provides valuable insight into early disease mechanisms.

Further interrogation of disease progression and the adaptive immune response would be possible allowing investigation of the interaction between innate and adaptive immune responses, albeit using protected live stages of zebrafish. Adult zebrafish have already been used successfully in corneal wound healing studies supporting the feasibility of such comparative approaches^71^.

In addition, the high regenerative capabilities inherent in zebrafish larvae which exceeds that of adult mammals, may influence both the kinetics and mechanisms of corneal wound healing observed in our study^72^. This regenerative potential is particularly pronounced in larval stages where tissue repair occurs with minimal scarring and often involves reactivation of developmental programs not readily accessible in adult mammals. Comparing healing responses across life stages could identify signalling pathways and cellular mechanisms that confer enhanced regenerative capacity in larvae versus adults. These insights may inform therapeutic strategies to enhance mammalian corneal regeneration.

Additionally as an aquatic organism, zebrafish lack the blinking mechanism and exposure to air that humans experience. This may influence the rate of wound healing observed but does not diminish the zebrafish’s value as a model system for studying a wide range of immunomodulatory pharmaceutical agents in a high-throughput manner, prior to transitioning promising candidates to other models—in line with the NC3R principles.

The bacterial bath approach used here provides a robust and reproducible means of eliciting inflammatory responses relevant to infectious exposure. Clinically swimming is a well-recognised risk factor for the development of MK^73^. While localised corneal inoculation would enable more precise interrogation of chemotactic gradients and immune-cell recruitment and was considered when establishing the model, maintaining a focal infection in zebrafish larvae presents technical challenges. These include rapid dilution of pathogens in the aquatic environment and the lack of protective mechanisms to retain the inoculum at the corneal surface. In mouse models, concentrated inocula of pathogens are added topically with or without retention strategies, such as eyelid closure (tarsorrhaphy) or contact lens placement^74,75^. Even in the absence of these retention strategies the pathogens tend to persist within the tear film, making them less susceptible to being washed away compared to the conditions found in our aquatic model. Future development of localised delivery methods may further enhance the translational relevance of the zebrafish corneal injury model.

We envision the eye injury and infection model described here to be useful in many ways. While we have utilised zebrafish lines with fluorescent immune cells further insights can be sought by investigating the different subtypes of immune cells. For example it has been demonstrated that macrophages are heterogenous with pro- and anti-inflammatory subtypes. These variants can be studied in further detail using lines which are fluorescent for tumour necrosis factor (TNF)^76^ or interleukin-1 (IL-1)^77^ for pro-inflammatory cells and arginase-2^78^ for anti-inflammatory cells. Integration of cytokine reporter lines and molecular analyses would directly link inflammatory signalling with cellular behaviour. This would further validate its relevance to human MK which is characterised by strong induction of pro-inflammatory cytokines IL-1β, TNF, IL-6 and neutrophil-attracting chemokines, such as IL-8/CXCL8^79^.

To further apply a reductionist approach, neutrophils or macrophages could be targeted for depletion to study their specific roles in the immune response and wound healing. This could be achieved using mutant lines lacking macrophages (*IRF8−/−*), or neutrophil with maturation-deficient lines (*Alas1−/−*), or the use of nitroreductase-expressing lines for temporal, promoter-specific ablation via metronidazole treatment^80–82^. Additionally post-injury, neutrophils and macrophages could be isolated using fluorescence-activated cell sorting allowing transcriptomic profiling investigation of differentially expressed genes in relation to injury and infection. Such analyses could reveal neutrophil phenotypic plasticity during the transition from inflammation to repair. Furthermore given the genetic tractability of zebrafish, various pathways of interest can be genetically manipulated in a variety of methods including CRISPR-Cas9^83^ or morpholinos, with short lag times^8^. Finally although we have focused on well-established bacteria, genetically modified pathogens^84^ can be employed to further study the effect of functional roles of bacterial genes^85,86^.

In summary, we describe a novel zebrafish larvae injury model, which we believe is a powerful model for studying the immune response in detail. This knowledge offers valuable opportunities to deepen our understanding of immunological pathways and to conduct high-throughput screening of immunomodulators. In addition, while ZIP has been designed for the eye injury model specifically, it can be utilised for a wide variety of injury purposes, particularly suited for high-precision or unilateral experimentation.

## Methods

### Zebrafish husbandry

We have complied with all relevant ethical regulations for animal use. All experiments were conducted under an approved UK Home Office Project Licence, by standards of humane animal care, as determined by the Animal (Scientific Procedures) Act UK 1986 and EU Directive 2010/63/EU in a UK Home Office-approved facility at the University of Edinburgh—the University of Edinburgh Bioresearch and Veterinary Service zebrafish facility at the Queen Medical Research Institute. Ethical approval for animal use was obtained from The University of Edinburgh Animal Welfare and Ethical Review Board; all experiments were undertaken under PPL P2394CA47, PP1017117 and PIL I57562145.

Unprotected zebrafish larvae defined as being less than 5 days post-fertilisation, were used in all experiments of this project. Transgenic zebrafish used for the experiments include *Tg(mpx:EGFP)*^21^, *Tg(mpeg1:mCherry)*^87^, *Tg(K19:GFP)*^88^, *Tg(LysC:mTurquoise)*^41^ and *Tg(mfap4:tdTomato-CAAX)*^42^. Fertilised zebrafish larvae were housed at 28.5 °C in conditioned water-reverse osmosis water supplemented with 60 µg/mL sea salt (Instant Ocean, Blacksburg, VA, USA) and imaged at room temperature (~22 °C) in 'fish water' (conditioned media supplemented with 4.2% (v/v) Tricaine to induce anaesthesia)^89^.

### Zebrafish larvae corneal injury model

A custom cast was designed using Tinkercad (accessed via https://www.tinkercad.com/) and printed using Formlabs Form 3 stereolithography 3D printer at a Z resolution of 0.025 mm to provide a bed to house the larvae to perform the injury (Fig. 1C). The cast was used to create impressions within heated 2% UltraPure™ Low Melting Point Agarose (ThermoFisher Scientific) and allowed to cool until the mould solidified.

The 3 dpf zebrafish larvae were anaesthetised in 4.2% (v/v) tricaine before the injury. Larvae were visualised using a Leica M205 stereomicroscope and positioned laterally within the ZIP wells with the left eye facing upwards using an Eppendorf GELoader 0.5–20 µL tip. Wells were kept filled with fish water when the larvae were not being injured. Just prior to injury, as much of the liquid in the well was removed. A 30G microlance needle was dipped in 20% ethanol before injuring the cornea. The boundaries of the cornea are delineated by the iris, which is pigmented. Ten effective scratches were performed per cornea. Effective corneal scraping was defined by the presence of cells visibly floating in the medium, indicating they had been abraded from the corneal surface. Larvae inflicted with excessive corneal injuries resulting in perforation of the cornea, defined by the visualisation of an opening within the iris surface or anterior luxation of the lens, and similarly ineffective corneal scraping, were excluded from the experiment.

### Bacterial sample preparation

*Pseudomonas aeruginosa* PA01 and *Staphylococcus aureus* ATCC25923 were used as a representative gram-negative and gram-positive bacterial species for this study, respectively. A single colony of bacteria was isolated from Luria Bertani (LB) Lennox agar plates and inoculated in 10 mL of fresh LB broth. This was incubated overnight in an orbital shaker at 250 rpm at 37 °C. One millilitre of the bacterial culture was transferred into 10 mL of fresh LB broth and incubated for 1 h at 37 °C. One millilitre of the subculture was centrifuged at 12,470 × *g* relative centrifugal force (× *g*) for 1 min and washed twice in sterile saline by centrifugation. The resultant pellet was resuspended in fish water with an optical density at 600 nm (OD_600_) of 0.01. To determine colony-forming units (CFU) the bacterial samples were serially diluted 1:10 in saline over eight dilutions and plated in triplicate (10 µL) onto LB agar. Plates were incubated overnight at 37 °C colonies were enumerated and presented as CFU mL^−1^. The concentration utilised was optimised to induce an inflammatory response without causing mortalities and the CFU count of the bacterial suspension that the larvae were immersed in averaged at 7 × 10^6^ CFU mL^−1^. To stain bacteria the bacterial subculture was incubated in CellVue Claret Far Red (Sigma- Aldrich) for 1 h before foetal bovine serum was added for 5 min. The resulting bacterial suspension was then washed with sterile saline before diluted at the final concentration in fish water.

### Bath immersion of zebrafish larvae with mediators and pathogen

Injured zebrafish larvae were transferred immediately after injury into ZIP wells with one larva per well containing 1 mL of fish water supplemented with vehicle control, inflammatory mediators (LPS from *Pseudomonas aeruginosa* (Sigma-Aldrich), fMLF (Sigma-Aldrich) or LTB_4_ (Cayman Chemicals)) or bacterial samples. Vehicle control was DMSO (Corning) or ethanol (Sigma-Aldrich), dependent on the type of mediator and recommended diluent. The concentration of the vehicle was matched to the mediator concentration used in the intervention group.

### Live imaging

To study gross neutrophil and macrophage migration, the embryos were imaged using the Leica M205 stereomicroscope at various time points. For time-lapses and detailed study of neutrophil and macrophage migratory behaviours, zebrafish larvae were mounted onto moulds created within individual wells of 96-well black, optically clear bottom plate (ViewPlate-96, Revvity). The moulds were formed using 2% UltraPure™ Low Melting Point Agarose (ThermoFisher Scientific) following the design of ZIP. The larvae were imaged using the Opera Pheonix Plus High-Content spinning disc confocal microscope. A Z-stack of 15 slices spaced at 2 µm were taken with the bottom Z-stack image taken where the green fluorescence of the basal corneal epithelium was detected. Images were taken every 20 s for 1.5 h. The excitation wavelengths of 405-, 480-, 587- and 647 nm were used to visualise CFP, EGFP, mCherry/tdTomato and CellVue Claret Far Red, respectively.

### Sample preparation for scanning electron microscopy

At designated time points, injured zebrafish larvae were fixed in 3% glutaraldehyde in 0.1 M sodium cacodylate buffer at pH 7.3 for 2 h. These were then washed trice in 0.1 M sodium cacodylate at 10-min intervals. The samples were subsequently post-fixed in 1% osmium tetroxide in 0.1 M sodium cacodylate for 45 min followed by three washes in 0.1 M sodium cacodylate at 10-min intervals. Dehydration of the sample was performed by immersion in graded acetone concentrations (50%, 70%, 90% and 100%) three times for 15 min each. The samples were then critical point dried using liquid carbon dioxide after which the samples were mounted on aluminium stubs with carbon tabs attached and sputter coated with 20 nm gold palladium. The samples were viewed using the Zeiss Crossbeam 550 focused ion beam scanning electron microscope.

### Data analysis and statistical analysis

Images acquired using LAS (Leica) were processed using ImageJ (Fiji) (National Institutes of Health, Bethesda). The number of inflammatory cells that infiltrated the cornea was manually counted. The TrackMate plugin was utilised to manually track fluorescent neutrophils and macrophages of z-stacks compiled as maximum intensity projection images^90^. Three-dimensional volumetric surface reconstructions of time-lapse images were generated using IMARIS (Version 10.2, Oxford Instruments). Bacteria were identified using the 'spots' function, whereas neutrophils, macrophages, and basal epithelial cells were segmented using the 'surfaces' function based on fluorescence intensity. A minimum diameter threshold of 1.5 µm was applied for bacterial identification.

GraphPad Prism Version 10.2.3 (GraphPad Software, San Diego, CA, USA) was utilised for graphing and statistical analyses. Two-way ANOVA with post-hoc analysis using Bonferroni’s multiple comparisons test and unpaired t-tests were used to analyse the results.

### Reporting summary

Further information on research design is available in the Nature Portfolio Reporting Summary linked to this article.

## Supplementary information

Supplementary Information

Description of Additional Supplementary Files

Supplementary Data 1

Supplementary Movie 1

Supplementary Movie 2

Supplementary Movie 3

Supplementary Movie 4

Reporting Summary

Transparent Peer Review File

## Data Availability

All Source data are provided with this paper in Supplementary Data 1. ZIP is available for download from Figshare^54^.
