# Supplementary material for: Real-time characterisation of microbe-induced inflammation using a novel zebrafish larval corneal injury and infection model
Source: Commun Biol. 2026 Apr 14;9:794. doi: 10.1038/s42003-026-09985-1 (PMC13261116; doi:10.1038/s42003-026-09985-1)
Supplement: Supplementary file 1 — Supplementary Information [file 42003_2026_9985_MOESM1_ESM.pdf]

Supplementary Figures

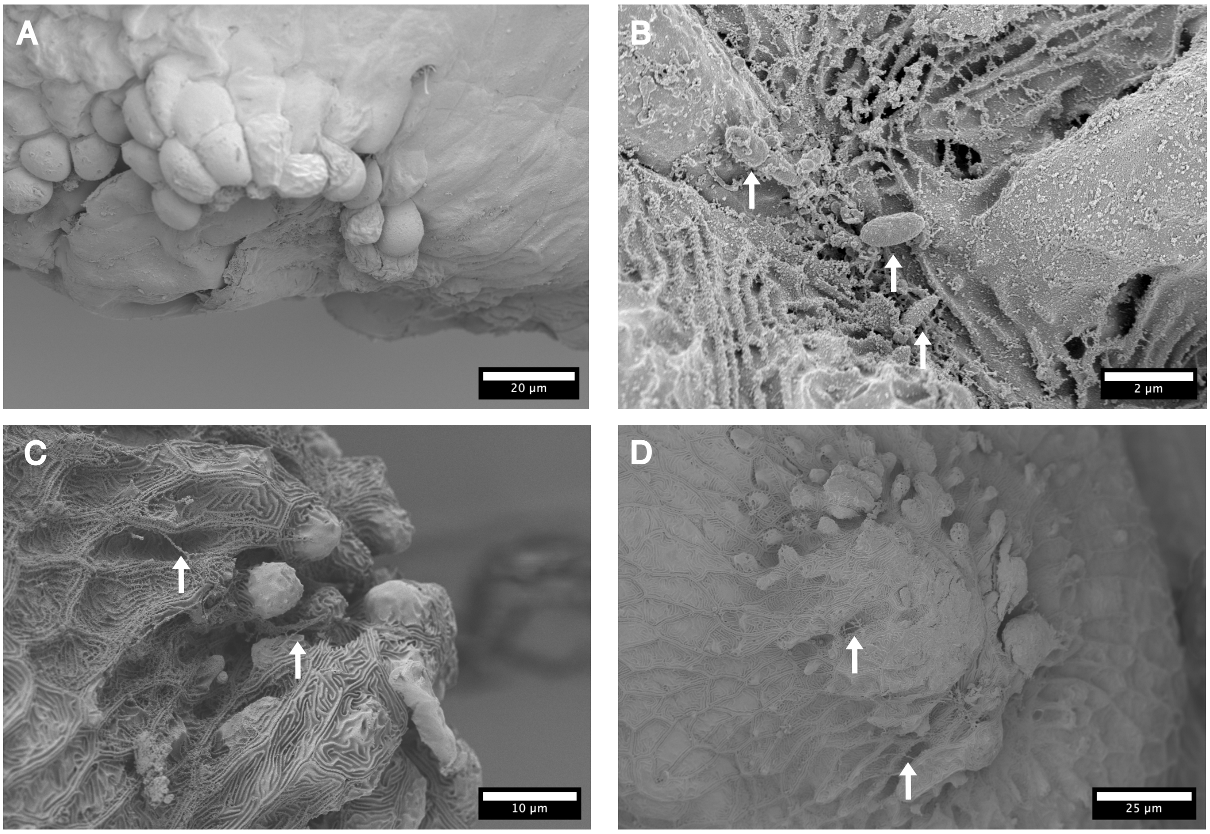

Supplementary Figure 1: *Scanning electron microscopy images of injured zebrafish larvae corneas.*

(A) Wounded cornea revealing underlying corneal stroma.

(B) Representative image of a wounded zebrafish cornea following immersion in a *P. aeruginosa* bacterial bath. Rod-shaped bacteria approximately 1 *µ*m in length (white arrows), consistent with *P. aeruginosa*, are visible within crevices of the injured cornea.

(C and D) Web-like structures (white arrows) seen within healing wound akin to fibrin or extracellular traps. Scale bar = 10 µm (C) and 25 µm (D).

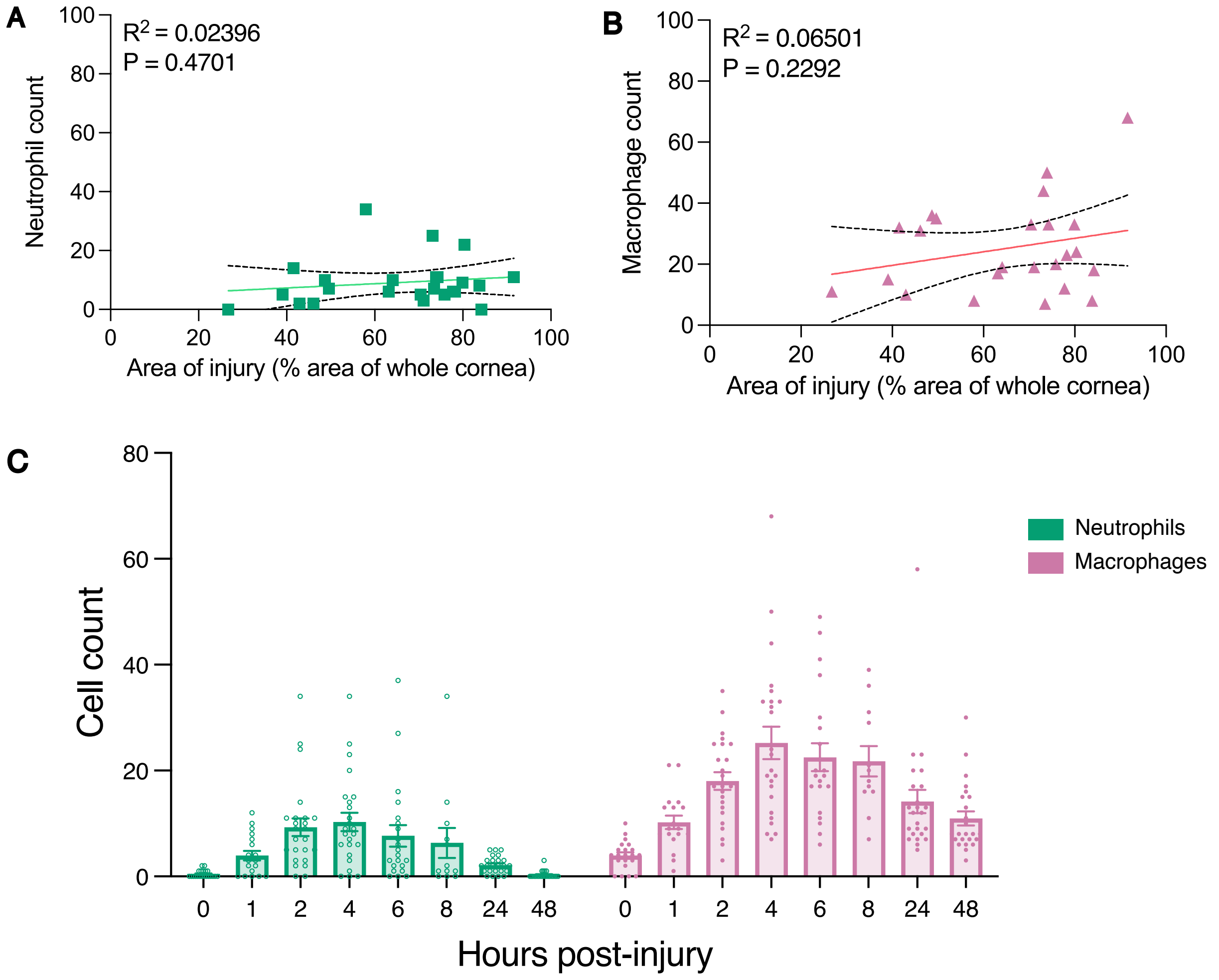

Supplementary Figure 2: *Validation of zebrafish larvae corneal injury model.*

(A) Correlation between area of corneal injury and neutrophil infiltration at the peak of infiltration for neutrophils (2 hpi). n=38 cornea in injured group and n=14 cornea in uninjured group, across three independent experiments.

(B) Correlation between area of corneal injury and macrophage infiltration at the peak of infiltration for macrophages (4 hpi). n=38 cornea in injured group and n=14 cornea in uninjured group, across three independent replicates.

(C) Quantification of neutrophil and macrophage cell number within the injured cornea in a Tg(K19:GFP; LysC:mTurquoise; mfap4:tdTomato-CAAX) zebrafish line. n=14 in each group, obtained over 4 separate independent experiments.

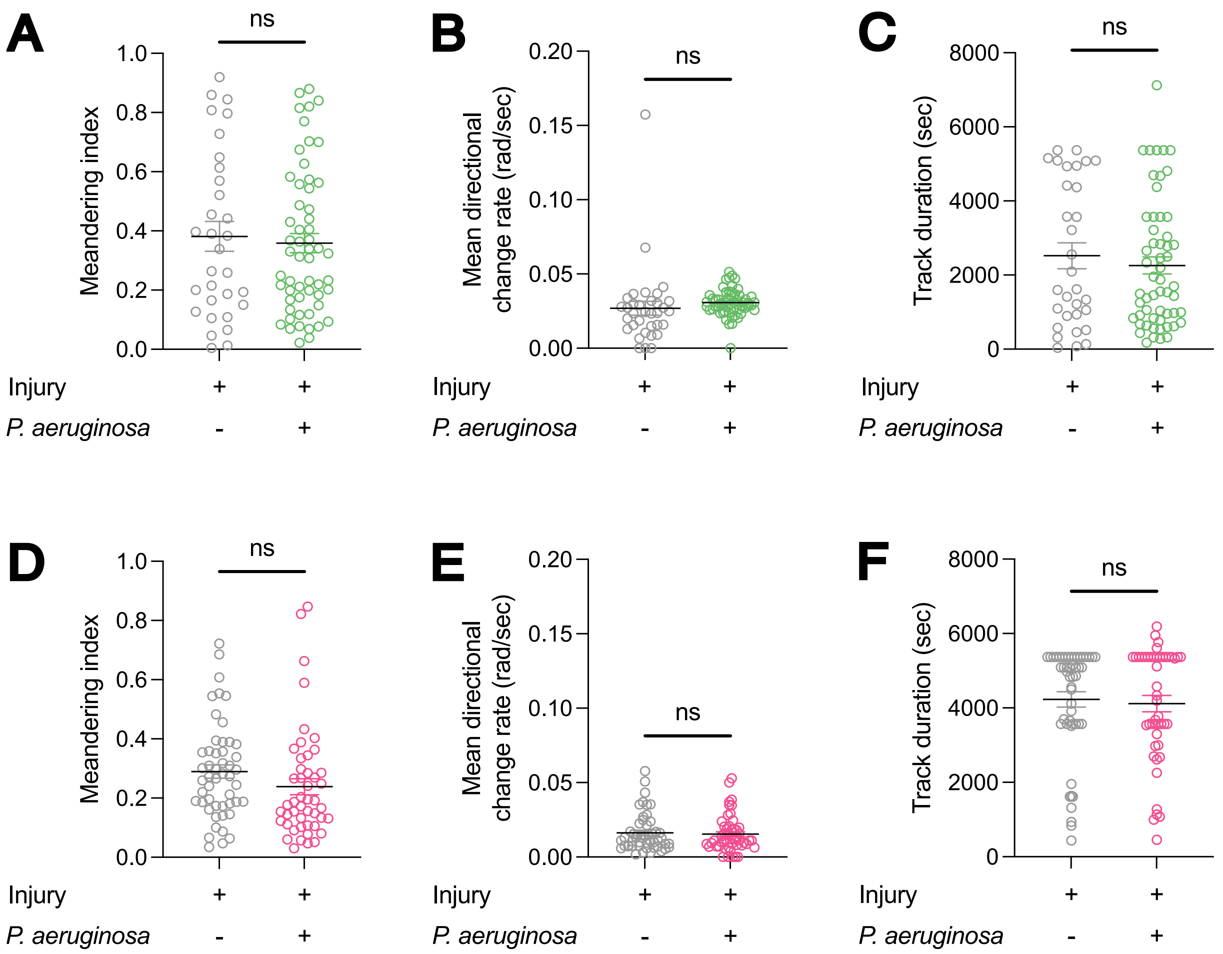

Supplementary Figure 3: Indices of motility of neutrophils and macrophages in sterile injury with or without infection with P. aeruginosa.

Quantification of neutrophil (A) meandering index, (B) mean directional change rate and (C) total track duration. (n= 31 in sterile injury group and 56 in injured and infected group.)

Quantification of macrophage (D) meandering index, (E) mean directional change rate and (F) total track duration. (n= 50 in sterile injury group and 52 in injured and infected group.)

Cells observed in 5 injured and 6 injured and infected larvae from 4 separate experiments. Unpaired T-tests. Error bars denote mean ± SEM. *$P \leq 0.05$; **$P \leq 0.01$; ***$P \leq 0.001$ and ****$P \leq 0.0001$.
