# Supplementary material for: Real-time characterisation of microbe-induced inflammation using a novel zebrafish larval corneal injury and infection model
Source: Commun Biol. 2026 Apr 14;9:794. doi: 10.1038/s42003-026-09985-1 (PMC13261116; doi:10.1038/s42003-026-09985-1)
Supplement: Supplementary file 2 — Description of Additional Supplementary Files [file 42003_2026_9985_MOESM2_ESM.docx]

**Description of Additional Supplementary Files**

**File name: Supplementary Data 1**

**Description:** The source data behind the graphs in the paper.

***File name: Supplementary movie 1***

***Description:*** *Widefield timelapse of a Tg(K19:GFP; LysC:mTurquoise) zebrafish larvae post-injury demonstrating rapid wound closure followed by accumulation of neutrophils within the cornea. Basal epithelial cells are fluorescent green while neutrophils are fluorescent red. Images taken every minute, total imaging time is 30 min. Scale bar = 250 µm.*

***File name: Supplementary movie 2***

***Description:*** *Maximum intensity projection timelapse of a Tg(K19:GFP, LysC:mTurquoise; mfap4: tdTomato-CAAX) zebrafish larvae post-injury demonstrating rapid wound closure, followed by accumulation of neutrophils and macrophages within the cornea. Basal epithelial cells are green, neutrophils are blue and macrophages are magenta. Images taken every 20 s, total imaging time is 29 min 40s. Scale bar = 100 µm.*

***File name: Supplementary movie 3***

***Description:*** *Three-dimensional reconstruction of injured and cornea of 3 day-post-fertilisation larvae infected with P. aeruginosa at 1 h post-infection demonstrating distribution of P. aeruginosa in relation to injured cornea and phagocytosis of bacteria by macrophages and neutrophils.* *Basal epithelial cells (green – transgene K19:EGFP), neutrophils (blue – transgene LysC:mTurquoise), macrophages (magenta – transgene mfap4:tdTomato-CAAX) and P. aeruginosa (yellow – labelled with CellVue Claret Far Red).*

***File name: Supplementary movie 4***

***Description:*** *Representative timelapse of injured corneas at 1 h-post injury with or without infection. 1.5 h timelapse (shown here as Maximum Intensity Projection) of a 3dpf larvae, showing basal epithelial cells (green – transgene K19: EGFP), neutrophils (blue – transgene LysC:mTurquoise), macrophages (magenta – transgene mfap4:tdTomato-CAAX) and P. aeruginosa (yellow – labelled with CellVue Claret Far Red).*
