# Supplementary material for: Real-time characterisation of microbe-induced inflammation using a novel zebrafish larval corneal injury and infection model
Source: Commun Biol. 2026 Apr 14;9:794. doi: 10.1038/s42003-026-09985-1 (PMC13261116; doi:10.1038/s42003-026-09985-1)
Supplement: Supplementary file 8 — Reporting Summary [file 42003_2026_9985_MOESM8_ESM.pdf]

Corresponding author(s): Dr Kelvin KW Cheng

Last updated by author(s): Jan 18, 2026

Reporting Summary

Nature Portfolio wishes to improve the reproducibility of the work that we publish. This form provides structure for consistency and transparency in reporting. For further information on Nature Portfolio policies, see our Editorial Policies and the Editorial Policy Checklist.

Statistics

For all statistical analyses, confirm that the following items are present in the figure legend, table legend, main text, or Methods section.

n/a	Confirmed	
☐	☒	The exact sample size (*n*) for each experimental group/condition, given as a discrete number and unit of measurement
☐	☒	A statement on whether measurements were taken from distinct samples or whether the same sample was measured repeatedly
☐	☒	The statistical test(s) used AND whether they are one- or two-sided<br>*Only common tests should be described solely by name; describe more complex techniques in the Methods section.*
☒	☐	A description of all covariates tested
☐	☒	A description of any assumptions or corrections, such as tests of normality and adjustment for multiple comparisons
☐	☒	A full description of the statistical parameters including central tendency (e.g. means) or other basic estimates (e.g. regression coefficient) AND variation (e.g. standard deviation) or associated estimates of uncertainty (e.g. confidence intervals)
☐	☒	For null hypothesis testing, the test statistic (e.g. *F*, *t*, *r*) with confidence intervals, effect sizes, degrees of freedom and *P* value noted<br>*Give P values as exact values whenever suitable.*
☒	☐	For Bayesian analysis, information on the choice of priors and Markov chain Monte Carlo settings
☒	☐	For hierarchical and complex designs, identification of the appropriate level for tests and full reporting of outcomes
☒	☐	Estimates of effect sizes (e.g. Cohen's *d*, Pearson's *r*), indicating how they were calculated

Our web collection on statistics for biologists contains articles on many of the points above.

Software and code

Policy information about availability of computer code

Data collection	Images were taken using a Leica M205 stereomicroscope or Opera Pheonix Plus High-Content spinning disc microscope.
Data analysis	Data were analysed using GraphPad Prism Version 10.2.3 (GraphPad Software, San Diego, CA, USA) and ImageJ (Fiji) (National Institutes of Health, Bethesda).

For manuscripts utilizing custom algorithms or software that are central to the research but not yet described in published literature, software must be made available to editors and reviewers. We strongly encourage code deposition in a community repository (e.g. GitHub). See the Nature Portfolio guidelines for submitting code & software for further information.

Data

Policy information about availability of data

All manuscripts must include a data availability statement. This statement should provide the following information, where applicable:

- Accession codes, unique identifiers, or web links for publicly available datasets
- A description of any restrictions on data availability
- For clinical datasets or third party data, please ensure that the statement adheres to our policy

All source data are provided with this paper. ZIP is available for download from Figshare (54).

Research involving human participants, their data, or biological material

Policy information about studies with human participants or human data. See also policy information about sex, gender (identity/presentation), and sexual orientation and race, ethnicity and racism.

Reporting on sex and gender	n/a
Reporting on race, ethnicity, or other socially relevant groupings	n/a
Population characteristics	n/a
Recruitment	n/a
Ethics oversight	n/a

Note that full information on the approval of the study protocol must also be provided in the manuscript.

Field-specific reporting

Please select the one below that is the best fit for your research. If you are not sure, read the appropriate sections before making your selection.

☒ Life sciences ☐ Behavioural & social sciences ☐ Ecological, evolutionary & environmental sciences

For a reference copy of the document with all sections, see nature.com/documents/nr-reporting-summary-flat.pdf

Life sciences study design

All studies must disclose on these points even when the disclosure is negative.

Sample size	Sample size was determined and calculated based on previous experience and pilot experiments in the Rossi laboratory.
Data exclusions	All data was included in analyses.
Replication	Experiments were replicated as detailed in figure legends.
Randomization	Larvae from each clutch were randomly selected when divided into groups.
Blinding	All experiments were performed with a large number of randomly assigned animals over many experimental replicates to reduce the risk of any unintended bias. When investigating the relationship between area of injury and immune cell recruitment, images of wounded cornea and immune cells were analysed blindly.

Reporting for specific materials, systems and methods

We require information from authors about some types of materials, experimental systems and methods used in many studies. Here, indicate whether each material, system or method listed is relevant to your study. If you are not sure if a list item applies to your research, read the appropriate section before selecting a response.

Materials & experimental systems

n/a	Involved in the study	
☒	☐	Antibodies
☒	☐	Eukaryotic cell lines
☒	☐	Palaeontology and archaeology
☐	☒	Animals and other organisms
☒	☐	Clinical data
☒	☐	Dual use research of concern
☒	☐	Plants

Methods

n/a	Involved in the study	
☒	☐	ChIP-seq
☒	☐	Flow cytometry
☒	☐	MRI-based neuroimaging

Animals and other research organisms

Policy information about studies involving animals; ARRIVE guidelines recommended for reporting animal research, and Sex and Gender in Research

Laboratory animals	Zebrafish (Danio rerio) up to 5 days post-fertilisation.<br>Transgenic lines used: Tg(mpx:EGFP; MPEG1:mCherry); Tg(K19:GFP; LysC:mTurquoise; mfap4:tdTomato-CAAX)
Wild animals	No wild animals were used in this study.
Reporting on sex	Sex was not considered in the design of the experiments as 3-5 day post fertilisation larvae have not undergone sexual differentiation at this stage and hence are indistinguishable. Larvae were selected at random for all experimental procedures.
Field-collected samples	No field collected samples were utilised in this project.
Ethics oversight	All experiments were conducted with the approval of the UK Home Office under the Project and Personal Licences PPL P2394CA47, PP1017117 and PIL I57562145, following standards of humane animal care, as determined by the Animal (Scientific Procedures) Act UK 1986 and EU Directive 2010/63/EU in a UK Home Office-approved facility, the University of Edinburgh Bioresearch and Veterinary Service zebrafish facility at the Queen Medical Research Institute.

Note that full information on the approval of the study protocol must also be provided in the manuscript.

Plants

Seed stocks	n/a
Novel plant genotypes	n/a
Authentication	n/a
