# Supplementary material for: Real-time characterisation of microbe-induced inflammation using a novel zebrafish larval corneal injury and infection model
Source: Commun Biol. 2026 Apr 14;9:794. doi: 10.1038/s42003-026-09985-1 (PMC13261116; doi:10.1038/s42003-026-09985-1)
Supplement: Supplementary file 9 — Transparent Peer Review File [file 42003_2026_9985_MOESM9_ESM.pdf]

nature portfolio

Peer Review File

Real-time characterisation of microbe-induced inflammation using a novel zebrafish larval corneal injury and infection model

Corresponding Author: Dr Kelvin Cheng

This file contains all reviewer reports in order by version, followed by all author rebuttals in order by version.

Version 0:

Reviewer comments:

Reviewer #1

(Remarks to the Author)
This manuscript describes the development and characterization of a novel zebrafish larval model for Microbial Keratitis (MK). The authors introduce a bespoke "Zebrafish Injury Platform" (ZIP) to standardize corneal injury and utilize transgenic reporter lines to visualize innate immune dynamics in real-time. The study comprehensively characterizes the recruitment of neutrophils and macrophages under conditions of sterile injury, pro-inflammatory mediator exposure, and bacterial infection. Notably, the authors identify distinct motility patterns, specifically increased neutrophil speed during infection. The strength of this manuscript is the new platform that the authors developed, which allows consistent injury and higher throughput to study corneal injury in zebrafish. There are several issues need to be addressed:
1. To facilitate the adoption of this model by the community, please consider providing the 3D printing design files (e.g., .stl files) for the ZIP mould in the Supplementary Materials or a repository link.
2. The observation that P. aeruginosa infection increases neutrophil speed (Figure 5) is intriguing. Do the authors have any hypothesis or preliminary data regarding the mechanism? Is it driven by specific bacterial virulence factors or host chemokine gradients? A brief discussion on this would add depth.

Reviewer #2

(Remarks to the Author)
“Real-time Characterisation and modulation of microbe-induced inflammation and resolution, using a novel zebrafish larval corneal injury and infection model”

In the present paper, authors developed an innovative zebrafish larval model for investigating the role of innate immune cells during corneal healing. By combining corneal injury with transgenic lines expressing fluorescent neutrophils, macrophages and basal epithelial cells, the model enables real-time, high-resolution visualization of immune cell dynamics. The authors show that corneal damage rapidly triggers leukocyte recruitment, which is further enhanced by pro-inflammatory mediators, and that infection with live bacteria induces a robust and sustained inflammatory response characterized by increased neutrophil speed and migratory distance. Overall, this platform provides a potentially powerful and scalable tool to support the discovery and screening of novel immunomodulators for microbial keratitis.
In my opinion, although the zebrafish model presented is interesting and unique in its approach, additional experimental data and text revisions or improvements would better support the reported findings and significantly strengthen the overall impact of the study.

Introduction
- I would recommend that the authors streamline and reduce the first page (page 3), in particular the section related to antibiotic resistance, which feels somewhat peripheral to the specific aims of the study. Instead, it might be more effective to focus on what is currently known about innate immune responses in the zebrafish cornea and on the role of immune cells during corneal regeneration, highlighting the key homologies with other vertebrates or human. This would better contextualize the novelty and relevance of the proposed model.

Results

- Supplementary Figure 1B does not appear to be referenced in the Results section. In addition, the authors should clarify how the identity of the bacteria shown in the SEM image was verified and explain the purpose of including this panel. If the SEM image represents the P. aeruginosa strain used in the infection experiments, this should be clearly stated in the figure legend; otherwise, the rationale for presenting it should be reconsidered or placed alongside the relevant experimental data.

- In Figure 3A, in the last lane (Injured + fMLF condition), the fluorescence images appear to show a marked increase in green cells (neutrophils) with comparatively few red cells (macrophages). However, in Figures 3F and 3G, quantification suggests a stronger increase in macrophages. This quantitative discrepancy is confusing, and I would recommend clarifying whether the representative image accurately reflects the quantified dataset. Clearer explanation or additional representative images may help the reader interpret these findings.

- To further support the relevance of the model to human microbial keratitis, the authors could consider assessing the expression of key pro-inflammatory cytokines known to be upregulated in this condition. In human MK, mediators such as IL-1β, TNF-α, IL-6, and particularly the neutrophil-attracting chemokine IL-8/CXCL8 are strongly induced following corneal infection and epithelial damage. Evaluating the zebrafish equivalents of these cytokines would provide an additional layer of biological validation, showing that the inflammatory pathways activated in this model parallel those observed in human disease.

Discussion
- Since fMLF and LTB4 are cited in previous zebrafish studies and are indeed known pro-inflammatory mediators commonly used in this model organism, it would be helpful if the authors could clarify whether these molecules are specifically relevant to corneal inflammation or to the pathological conditions for which this model is intended. Are these factors analyzed ideals? Or is it possible to verify the cell behavior using more appropriate factors, which offer a closer parallel to the clinical scenario. Clarifying this aspect would improve the understanding of the translational relevance of the selected stimuli.

- In the Discussion, it would be helpful if the authors could clarify the rationale for using the Tg(LysC:mTurquoise) and Tg(mfap4:tdTomato-CAAX) lines for the cell motility analyses, rather than relying solely on the Tg(mpx:EGFP) and Tg(mpeg1:mCherry) lines used throughout the main injury and infection experiments. Although LysC and mfap4 are established markers of myeloid cells, it would be valuable to explain why these alternative reporters were selected for the speed measurements and what advantages they offer in this context. An explanation would help readers better understand the choice of transgenic combinations and how these lines complement each other within the study.

- Since young zebrafish larvae do not yet possess a fully developed adaptive immune system, it would be useful to address how this limitation may influence the interpretation of the model, especially considering that adaptive immunity contributes to later stages of microbial keratitis in humans. It could be possible to consider the use of this model also in the subsequent developmental stages, where adaptive immune response is established?

Reviewer #3

(Remarks to the Author)
In this manuscript, Cheng et al. present a needle-scratch based corneal injury model to assess immune activation and inflammation in the zebrafish eye as an approach to study microbial keratitis (MK). By leveraging the optical transparency of zebrafish and fluorescently labeled basal epithelial cells, macrophages, and neutrophils, the authors aim to characterize immune-cell recruitment, resolution, and tissue repair in their MK framework. While their newly designed injury platform is an addition to consistency and ease of execution to the field. This is indeed a creative model that capitalizes on the strengths of zebrafish. To help further strengthen the depth and impact of this work, I offer the following suggestions.

The authors note the presence of web-like structures at 15 minutes post-injury and speculate they may represent fibrin clots or NETs. However, given the reported kinetics showing minimal neutrophil recruitment at this early time point the origin of these structures is unclear. Clarifying how sterile injury could generate NETs, or providing supporting evidence, would improve confidence in this interpretation. In addition, incorporating a TGF-β reporter line could offer valuable insights into immune resolution and wound-healing dynamics.

It may also be informative to explore whether neutrophils directly contribute to corneal repair. Approaches such as transient neutrophil depletion, blocking migration, or profiling sorted injury-recruited neutrophils could help determine whether matrix-producing or pro-resolution genes are activated.

To enhance the relevance of this model to infectious MK, the authors might consider complementing the current bacterial bath approach with a more localized corneal infection model such as using a needled dipped in bacterial solution or slowly releasing a small volume of bacteria while scratching. This would enable a more faithful assessment of immune-cell recruitment and chemotactic signaling compared with whole body bacterial exposure or chemoattractant treatment alone. Given the unique aspects of zebrafish corneal biology, it would also be helpful for the authors to address model-specific limitations such as regenerative capacity, developmental growth dynamics, and differences in dominant photoreceptor composition and how these factors may influence interpretation relative to mammalian systems.

Because one major advantage of the zebrafish system is the ability to track directed cell migration, it would strengthen the manuscript and the resolution of MK to more fully describe how inflammatory cells dissipate after recruitment. For example, do cells undergo reverse migration, random dispersion, or cell death? Since imaging extends to two days post-injury, the

authors could consider using a Dendra2 photoconversion line to distinguish newly recruited cells from longer-resident "aged" neutrophils.

Finally, the manuscript would benefit from a more explicit comparison of this MK model to mammalian systems and models, particularly regarding immune-cell kinetics and the overall inflammatory trajectory.

Version 1:

Reviewer comments:

Reviewer #1

(Remarks to the Author)
The authors have addressed all my questions, and have made the necessary changes. I have no further comments.

Reviewer #2

(Remarks to the Author)
Dear authors,
Following careful evaluation of the responses to all my previous comments, as well as the revised text, figures, and the additional data included in the manuscript, I find that the manuscript has been significantly improved. The study is now more complete and, in its current form, I consider it suitable for publication. I particularly appreciate the authors' choice to propose an alternative experimental model which, despite some inherent limitations, proves to be a valuable and functional tool for the study of important biological processes and has the potential to provide insights that are also relevant to human health.

Reviewer #3

(Remarks to the Author)
The authors have addressed my comments via discussion.

Open Access This Peer Review File is licensed under a Creative Commons Attribution 4.0 International License, which permits use, sharing, adaptation, distribution and reproduction in any medium or format, as long as you give appropriate credit to the original author(s) and the source, provide a link to the Creative Commons license, and indicate if changes were made.
In cases where reviewers are anonymous, credit should be given to 'Anonymous Referee' and the source.
The images or other third party material in this Peer Review File are included in the article's Creative Commons license, unless indicated otherwise in a credit line to the material. If material is not included in the article's Creative Commons license and your intended use is not permitted by statutory regulation or exceeds the permitted use, you will need to obtain permission directly from the copyright holder.
To view a copy of this license, visit https://creativecommons.org/licenses/by/4.0/

Adriano Rossi BSc, PhD, DSc, FRSB, FBPhS
Professor of Respiratory and Inflammation Pharmacology
Deputy Director of the Centre for Inflammation Research
The University of Edinburgh
Institute for Regeneration and Repair
4-5 Little France Drive
Edinburgh EH16 4UU

E: adriano.rossi@ed.ac.uk
Secretary: E: sonia.rafferty@ed.ac.uk

18th January 2026

Manuscript Reference ID: COMMSBIO-25-11177-T

Manuscript Title: Real-time characterisation and modulation of microbe-induced inflammation and resolution, using a novel zebrafish larval corneal injury and infection model

Dear Editor,

Thank you for reviewing our manuscript and for the constructive comments. We appreciate the time taken to provide these comments and feel that the changes have helped to significantly improve the manuscript. We have also updated the graphs to comply with editorial requirements and supplied the raw data files in Figshare. Please see our response to the reviewers' comments below.

Reviewer #1:

Thank you for your positive and encouraging review of our work.

1. ***To facilitate the adoption of this model by the community, please consider providing the 3D printing design files (e.g., .stl files) for the ZIP mould in the Supplementary Materials or a repository link.***

Thank you for the comment. The .stl files for the ZIP moulds has now been uploaded to Figshare and referenced in the Results section.

Line 473-474:

To facilitate adoption of the model, the ZIP 3D printing design file is available from Figshare (54).

2. *The observation that P. aeruginosa infection increases neutrophil speed (Figure 5) is intriguing. Do the authors have any hypothesis or preliminary data regarding the mechanism? Is it driven by specific bacterial virulence factors or host chemokine gradients? A brief discussion on this would add depth.*

Thank you for the comment. We have several hypotheses to explain these findings including differences in chemotactic gradients, predominant chemokines involved (DAMPs vs PAMPs) and the effect of different bacterial strains on neutrophil responses. We have added the following to the discussion section to address this comment. This could certainly form the basis for further in-depth investigation in the future.

Lines 528-540:
We hypothesise that neutrophil speed is altered in response to several factors including greater chemokine gradients in infected tissues and types of chemokines released. Infections are typically characterised by the presence of pathogen-associated molecular patterns (PAMPS) while sterile injuries primarily involve the release of damage-associated molecular patterns (DAMPs). It is well established that neutrophils prioritise pathogen-derived factors such as complement factor 5a and formylated peptides compared to host-secreted-factors such as interleukin-8 and LTB4 (68). This is in agreement with Richardson et al., who showed that in vitro neutrophil speed and ROS production were differentially increased in response to specific strains of bacteria (69). In addition, specific strains of bacteria have been demonstrated to elicit different neutrophil responses with Streptococcal strains being potent inducers of heparin-binding protein and resistin, both reportedly associated with sepsis severity (70).

Reviewer 2:

Many thanks for your comments and positive evaluation of the manuscript.

1. I would recommend that the authors streamline and reduce the first page (page 3), in particular the section related to antibiotic resistance, which feels somewhat peripheral to the specific aims of the study. Instead, it might be more

effective to focus on what is currently known about innate immune responses in the zebrafish cornea and on the role of immune cells during corneal regeneration, highlighting the key homologies with other vertebrates or human. This would better contextualize the novelty and relevance of the proposed model.

Thank you for the comment. We have removed the section related to antibiotic resistance in the Introduction. As this is a novel model system, this is the first report of the innate immune response in the zebrafish cornea. However, we have added a section in the introduction highlighting the key homologies of zebrafish cornea to vertebrates and humans.

Lines 86-89:
The zebrafish cornea shares strong conservation of features both structurally, having similar layered organisation and possession of a distinct peripheral limbal stem cell niche like humans, and molecularly, with several molecular markers such as keratan sulfate and keratin 3 being expressed in both species (25).

2. Supplementary Figure 1B does not appear to be referenced in the Results section. In addition, the authors should clarify how the identity of the bacteria shown in the SEM image was verified and explain the purpose of including this panel. If the SEM image represents the *P. aeruginosa* strain used in the infection experiments, this should be clearly stated in the figure legend; otherwise, the rationale for presenting it should be reconsidered or placed alongside the relevant experimental data.

We apologise for this omission and have now referenced Supplementary Figure 1B in the Results section (line 318). The image was taken from a sample which was immersed in *P. aeruginosa*. Its morphological features (rod shaped bacteria of a length of approximately 1μm) are consistent with bacilli, supporting this identification. The figure legend has been updated to explicitly describe how this determination was made.

Supplementary Figure 1B legend:

Representative image of a wounded zebrafish cornea following immersion in a *P. aeruginosa* bacterial bath. Rod-shaped bacteria approximately 1 μm in length (white arrows), consistent with *P. aeruginosa*, are visible within crevices of the injured cornea.

3. In Figure 3A, in the last lane (Injured + fMLF condition), the fluorescence images appear to show a marked increase in green cells (neutrophils) with comparatively few red cells (macrophages). However, in Figures 3F and 3G, quantification suggests a stronger increase in macrophages. This quantitative discrepancy is confusing, and I would recommend clarifying whether the representative image accurately reflects the quantified dataset. Clearer explanation or additional representative images may help the reader interpret these findings.

Thank you for this observation. In the original version of Figure 3A, the red channel in the final lane appeared dim. The contrast has now been enhanced in the revised panel to improve visualisation. Macrophage numbers in the control and injured + fMLF groups are comparable, which is reflected in the quantification (Figure 3G). Although the injured + fMLF group shows a slightly higher mean, this difference was not statistically significant.

Updated Figure 3:

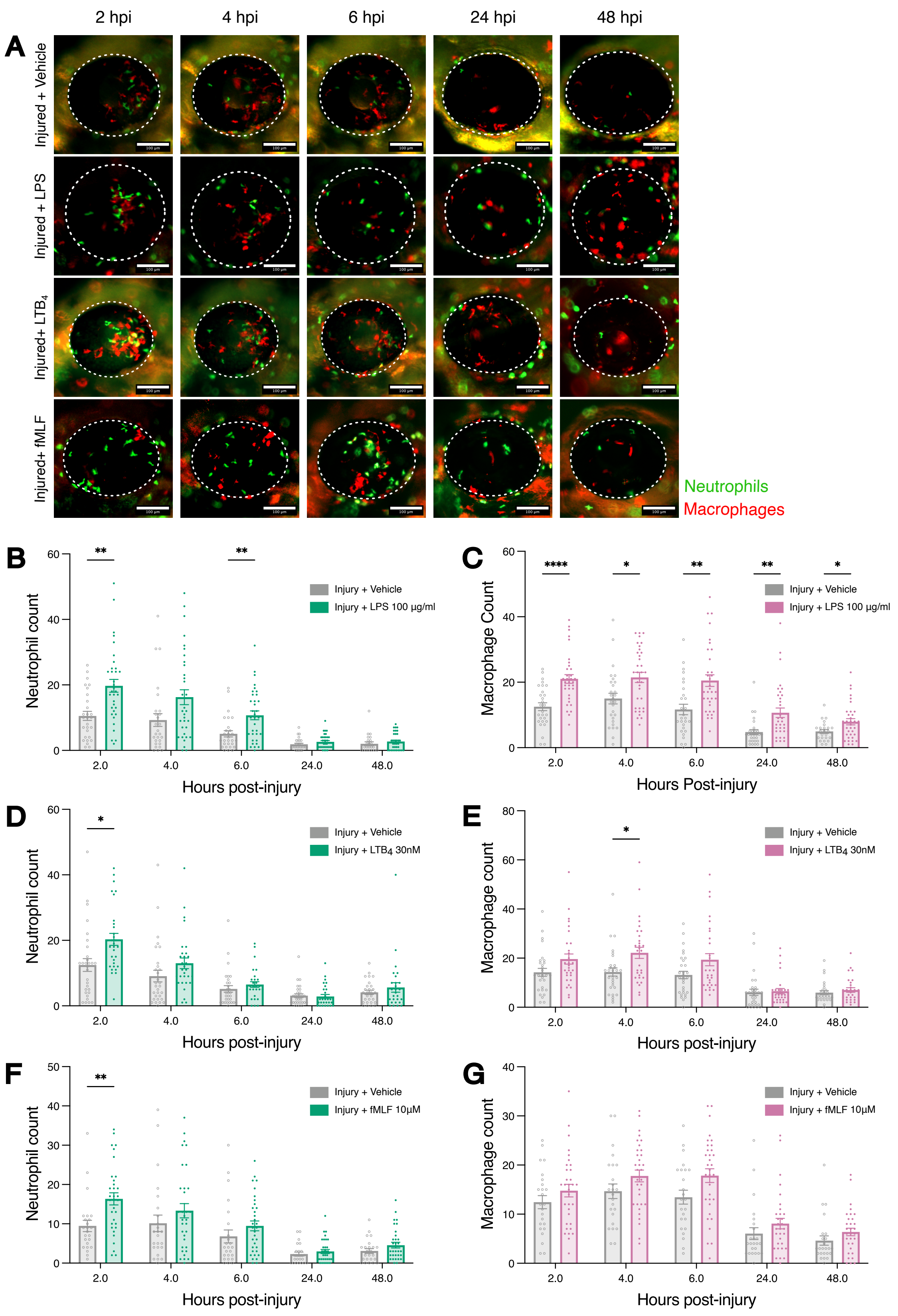

4. To further support the relevance of the model to human microbial keratitis, the authors could consider assessing the expression of key pro-inflammatory cytokines known to be upregulated in this condition. In human MK, mediators such as IL-1β, TNF-α, IL-6, and particularly the neutrophil-attracting chemokine IL-8/CXCL8 are strongly induced following corneal infection and epithelial

damage. Evaluating the zebrafish equivalents of these cytokines would provide an additional layer of biological validation, showing that the inflammatory pathways activated in this model parallel those observed in human disease.

Thank you for this helpful suggestion. We agree that evaluating pro-inflammatory cytokines relevant to human microbial keratitis, such as IL-1β, TNF, IL-6, and neutrophil-attracting chemokines, would further strengthen the biological relevance of the model. Zebrafish transgenic reporter lines for cytokines including TNF and IL-1β provide powerful tools for such analyses. We have therefore added the following in the discussion highlighting this as an important future direction.

Lines 597-605:
For example, it has been demonstrated that macrophages are heterogenous with pro- and anti-inflammatory subtypes. These variants can be studied in further detail using lines which are fluorescent for tumour necrosis factor (TNF) (76) or interleukin-1 (IL-1) (77) for pro-inflammatory cells and arginase-2 (78) for anti-inflammatory cells. Integration of cytokine reporter lines and molecular analyses would directly link inflammatory signalling with cellular behaviour. This would further validate its relevance to human MK, which is characterised by strong induction of pro-inflammatory cytokines IL-1β, TNF, IL-6, and neutrophil-attracting chemokines such as IL-8/CXCL8 (79).

5. Since fMLF and LTB4 are cited in previous zebrafish studies and are indeed known pro-inflammatory mediators commonly used in this model organism, it would be helpful if the authors could clarify whether these molecules are specifically relevant to corneal inflammation or to the pathological conditions for which this model is intended. Are these factors analyzed ideals? Or is it possible to verify the cell behavior using more appropriate factors, which offer a closer parallel to the clinical scenario. Clarifying this aspect would improve the understanding of the translational relevance of the selected stimuli.

Thank you for raising this important point regarding the translational relevance of the inflammatory stimuli used. Both fMLF and LTB_4 are highly relevant mediators in bacterial keratitis, which is the primary pathological condition this model aims to recapitulate.

The University of Edinburgh is a charitable body, registered in Scotland, with registration number SC005336

fMLF represents a pathogen-associated molecular pattern released during bacterial infection and directs neutrophil chemotaxis via formyl peptide receptors *(Dorward et al. 2015, Mader et al. 2010).* Formyl peptides are produced by common keratitis-causing organisms, including *P. aeruginosa* and *S. aureus*, making fMLF a widely accepted proxy for bacterial-derived chemotactic signals in infection models.

LTB_4 is likewise clinically relevant on the ocular surface. Human corneal and conjunctival epithelial cells produce LTB_4 in response to bacterial lipopolysaccharide *(Sahin et al. 2012),* and elevated tear LTB_4 levels have been reported in contact lens-associated inflammatory conditions and allergic ocular disease *(Thakur et al. 1998).* Increased LTB_4 has also been documented in murine models of *Pseudomonas* *(Kernacki et al. 1995)* keratitis, where it correlates with neutrophil recruitment.

Thus, fMLF and LTB_4 are not generic inflammatory cues but key components of the bacterial keratitis inflammatory cascade. Their use allows controlled and reproducible interrogation of immune-cell behavior, complementing live infection models while minimizing experimental variability. We now clarify this rationale in the manuscript.

Lines 482-492:
These inflammatory mediators were selected for their established relevance to bacterial keratitis. Formyl peptides such as fMLF act as pathogen-associated molecular patterns released during bacterial infection and drive neutrophil chemotaxis via formyl peptide receptors (57, 58). LTB4 is a potent lipid chemoattractant produced by corneal epithelial cells in response to bacterial stimuli (59), is elevated in human tear fluid during ocular surface inflammation, including bacterial keratitis and contact lens–associated disease (60, 61) and correlated with neutrophil recruitment in mouse models of P. aeruginosa keratitis. Employing these mediators enables mechanistic dissection of immune-cell recruitment downstream of clinically relevant inflammatory signals while maintaining experimental control and reproducibility.

6. In the Discussion, it would be helpful if the authors could clarify the rationale for using the Tg(LysC:mTurquoise) and Tg(mfap4:tdTomato-CAAX) lines for the cell motility analyses, rather than relying solely on the Tg(mpx:EGFP) and Tg(mpeg1:mCherry) lines used throughout the main injury and infection experiments. Although LysC and mfap4 are established markers of myeloid cells, it would be valuable to explain why these alternative reporters were

selected for the speed measurements and what advantages they offer in this context. An explanation would help readers better understand the choice of transgenic combinations and how these lines complement each other within the study.

Thank you for this comment. The selection of Tg(LysC:mTurquoise) and Tg(mfap4:tdTomato-CAAX) for cell motility analyses was driven by technical requirements for multi-channel live imaging. Our experimental design necessitated simultaneous visualization of neutrophils, macrophages, and basal epithelial cells to comprehensively assess cellular dynamics during corneal inflammation. However, the Tg(mpx:EGFP) line used in our primary experiments labels neutrophils with GFP, which is spectrally incompatible with the Tg(K19:GFP) line marking basal epithelial cells. To circumvent this limitation, we employed alternative myeloid reporters: LysC (a well-established neutrophil marker) driving mTurquoise and mfap4 (a reliable macrophage marker) driving tdTomato-CAAX. This fluorophore combination eliminated spectral overlap and enabled us to simultaneously track immune cell recruitment and motility in relation to epithelial cell behavior. The following has been added to the results section for clarity:

Lines 227-240:

To enable comprehensive analysis of cellular interactions during corneal inflammation, which we will describe later, we required simultaneous visualization of basal epithelial cells, neutrophils, and macrophages. This necessitated the development of an alternative transgenic line with compatible fluorescent reporters. Since the Tg(K19:GFP) line labels basal epithelial cells with GFP, we could not use our standard Tg(mpx:EGFP) neutrophil reporter due to spectral overlap. To address this technical constraint, we generated the triple-transgenic line Tg(K19:GFP;LysC:mTurquoise;mfap4:tdTomato-CAAX), which employs LysC-driven mTurquoise for neutrophils (41) and mfap4-driven tdTomato-CAAX for macrophages (42). This fluorophore combination (GFP/mTurquoise/tdTomato) provided distinct spectral separation, allowing us to simultaneously track epithelial, neutrophil, and macrophage populations in the experiments. Here, we validate that this line with alternative promoters for neutrophils and macrophages show similar patterns of immune cell migration.

6. Since young zebrafish larvae do not yet possess a fully developed adaptive immune system, it would be useful to address how this limitation may influence the interpretation of the model, especially considering that adaptive immunity contributes to later stages of microbial keratitis in humans. It could be possible to consider the use of this model also in the subsequent developmental stages, where adaptive immune response is established?

Thank you for this insightful comment. We agree that addressing the limitations of the developing adaptive immune system is important for interpreting our model. However, the zebrafish larval model provides a unique advantage: it allows us to dissect the innate immune response in isolation, which is not readily achievable in other experimental systems. This is particularly valuable given that innate immunity plays a critical role in the early stages of microbial keratitis pathogenesis.

We acknowledge that adaptive immunity contributes to later disease stages in humans. As suggested, extending this model to adult zebrafish with fully developed adaptive immune systems represents a promising future direction. Previous studies have successfully utilized adult zebrafish for corneal wound healing investigations *(Ikkala et al., 2022),* demonstrating the feasibility of this approach. Comparative studies between larval and adult zebrafish could provide important insights into how innate immune responses are modulated in the presence of adaptive immunity, potentially revealing stage-specific therapeutic targets for microbial keratitis.

We have modified the discussion section to reflect this.

Lines 550-560:
While this restricts direct modelling of later stages of MK - where adaptive immunity contributes to disease progression - it also represents a strength of the system by enabling high-resolution analysis of innate immune responses in isolation. Given the critical role of innate immunity in initiating microbial keratitis, this approach provides valuable insight into early disease mechanisms.

Further interrogation of disease progression and the adaptive immune response would be possible, allowing investigation of interaction between innate and adaptive immune responses, albeit using protected live stages of zebrafish. Adult zebrafish have already

The University of Edinburgh is a charitable body, registered in Scotland, with registration number SC005336

been used successfully in corneal wound healing studies, supporting the feasibility of such comparative approaches (71).

Reviewer 3:

We are very grateful for the positive remarks, thoughtful comments and ideas to improve the manuscript.

1. The authors note the presence of web-like structures at 15 minutes post-injury and speculate they may represent fibrin clots or NETs. However, given the reported kinetics showing minimal neutrophil recruitment at this early time point the origin of these structures is unclear. Clarifying how sterile injury could generate NETs, or providing supporting evidence, would improve confidence in this interpretation. In addition, incorporating a TGF-β reporter line could offer valuable insights into immune resolution and wound-healing dynamics.

Thank you for the insightful comment about the NET-like structures observed at 15-mins post-injury and minimal neutrophil recruitment at this timepoint.

Regarding the possibility of NETs in sterile injury conditions, there is evidence that NET development is not solely dependent on bacterial induction but is also released in sterile inflammation *(Wang et al. 2024).* In sterile injuries, damage-associated molecular patterns (DAMPs) such as histones, mitochondrial DNA and HMGB1 released from damaged cells can trigger NET formation *(Kim et al. 2020; Tadie et al. 2013; Block et al. 2022).* Additionally, 'vital NETs', which do not require cell death, have been demonstrated to form rapidly, within 5-60 mins of stimulation with mediators such as C5a, unlike suicidal NETs which takes several hours *(Yousefi et al. 2009).* Indeed, these rapid NETs can also be mediated by TLR4 on platelets *(Yipp et al. 2013).* Hence, mechanistically, indeed this is possible. However, we agree with the reviewer that the current data are insufficient to definitely establish whether these structures represent NETs, early fibrin clots or a combination of both extracellular matrices. With this in mind, we have revised the text to acknowledge this uncertainty more explicitly.

We agree that utilizing the Smad3/ TGF-β reporter line, Tg(12xSBE:EGFP), represents a compelling avenue for investigating immune resolution and wound healing dynamics. While this line has previously been characterized primarily in the context of neural

differentiation, its application to post-injury recovery is highly relevant. We recognize its potential value for future research into the signaling pathways governing tissue repair. We have modified the results section to enhance clarity:

Lines 137-147:

This could represent a fibrin clot or the formation of neutrophil extracellular traps (NETs) (9, 28) or a combination of both, which requires further investigation. The NETs seen rapidly post-injury may represent vital NETs which have been reported to form rapidly, within five to 60 minutes post stimulation (29), by mediators such as C5a or TLR4 stimulation on platelets (30). We hypothesise that fibrin clots form rapidly and this could then stimulate the formation of vital NETs by recruited neutrophils. Nonetheless, these findings are preliminary and will require further characterisation. Additionally, studying post-injury tissue healing wound mechanics and immune resolution could be further deepened using the Smad3/ TGF-β reporter line (31) as TGF-β is important in mediating wound healing in most tissues, including the cornea (32-35).

2. It may also be informative to explore whether neutrophils directly contribute to corneal repair. Approaches such as transient neutrophil depletion, blocking migration, or profiling sorted injury-recruited neutrophils could help determine whether matrix-producing or pro-resolution genes are activated.

Thank you for this insightful comment. We agree these are interesting approaches and hence have included them in future directions as seen below:

Lines 607-615:

To further apply a reductionist approach, neutrophils or macrophages cold be targeted for depletion to study their specific roles in the immune response and wound healing. This could be achieved using mutant lines lacking macrophages (*IRF8-/-),* or neutrophil with maturation-deficient lines (*Alas1-/-),* or the use of nitroreductase-expressing lines for temporal, promoter-specific ablation via metronidazole treatment (80-82). Additionally, post-injury, neutrophils and macrophages could be isolated using fluorescence-activated cell sorting allowing transcriptomic profiling investigation of differentially expressed genes in relation to injury and infection. Such analyses could reveal neutrophil phenotypic plasticity during the transition from inflammation to repair.

3. To enhance the relevance of this model to infectious MK, the authors might consider complementing the current bacterial bath approach with a more localized corneal infection model such as using a needled dipped in bacterial solution or slowly releasing a small volume of bacteria while scratching. This would enable a more faithful assessment of immune-cell recruitment and chemotactic signaling compared with whole body bacterial exposure or chemoattractant treatment alone.

Thank you for this valuable suggestion. We agree that a localized corneal infection model would be ideal for modelling microbial keratitis and allow more precise assessment of immune-cell recruitment.

We initially considered delivering bacteria via a bacteria-dipped needle; however, this approach presents technical limitations in zebrafish larvae. Effective corneal scratching requires pre-dipping the needle in 20% ethanol, which would likely reduce bacterial viability and lead to inconsistent inoculation. In addition, larvae must be returned immediately to an aqueous environment post-injury, increasing the likelihood that locally applied bacteria would be rapidly diluted or washed away before infection can establish.

These challenges parallel those in mammalian keratitis models, where eyelid closure or contact lenses are required to retain the inoculum - approaches not feasible in a zebrafish system. Moreover, in mammals, a thin tear film maintains ocular surface moisture, reducing the extent of pathogen dilution. Swimming is a well-recognised risk factor for microbial keratitis, and thus exposure to a bacterial bath provides a biologically relevant surrogate for this scenario *(Shankar et al. 2012).*

With these factors in mind, we employed a bacterial bath as a reproducible and robust method to elicit inflammation while maintaining experimental consistency. We now acknowledge this limitation and note that future methodological advances may enable sustained localized infection in this model.

Lines 579-593:

The bacterial bath approach used here provides a robust and reproducible means of eliciting inflammatory responses relevant to infectious exposure. Clinically, swimming is a well-recognised risk factor for the development of MK (73). While localized corneal

inoculation would enable more precise interrogation of chemotactic gradients and immune-cell recruitment and was considered when establishing the model, maintaining a focal infection in zebrafish larvae presents technical challenges. These include rapid dilution of pathogens in the aquatic environment and the lack of protective mechanisms to retain the inoculum at the corneal surface. In mouse models, concentrated inocula of pathogens are added topically with or without retention strategies, such as eyelid closure (tarsorrhaphy) or contact lens placement (74, 75). Even in the absence of these retention strategies, the pathogens tend to persist within the tear film, making them less susceptible to being washed away compared to the conditions found in our aquatic model. Future development of localised delivery methods may further enhance the translational relevance of the zebrafish corneal injury model.

4. Given the unique aspects of zebrafish corneal biology, it would also be helpful for the authors to address model-specific limitations such as regenerative capacity, developmental growth dynamics, and differences in dominant photoreceptor composition and how these factors may influence interpretation relative to mammalian systems.

Thank you for this insightful comment. We have now elaborated on zebrafish-specific biological considerations and their implications for cross-species interpretation in the Discussion section.

Lines 562-570:

In addition, the high regenerative capabilities inherent in zebrafish larvae which exceeds that of adult mammals may influence both the kinetics and mechanisms of corneal wound healing observed in our study (72). This regenerative potential is particularly pronounced in larval stages, where tissue repair occurs with minimal scarring and often involves reactivation of developmental programs not readily accessible in adult mammals. Comparing healing responses across life stages could identify signalling pathways and cellular mechanisms that confer enhanced regenerative capacity in larvae versus adults. These insights may inform therapeutic strategies to enhance mammalian corneal regeneration.

5. Because one major advantage of the zebrafish system is the ability to track directed cell migration, it would strengthen the manuscript and the resolution of MK to more fully describe how inflammatory cells dissipate after recruitment.

For example, do cells undergo reverse migration, random dispersion, or cell death? Since imaging extends to two days post-injury, the authors could consider using a Dendra2 photoconversion line to distinguish newly recruited cells from longer-resident "aged" neutrophils.

Thank you for this thoughtful comment. We agree that determining the fate of recruited neutrophils, whether through reverse migration, dispersion, or cell death, is an important aspect of inflammation resolution and represents a strength of the zebrafish model. Use of a *Dendra2* photoconversion line would indeed allow long-term tracking of individual neutrophils and discrimination between newly recruited and longer-resident cells.

However, such lineage-tracking experiments fall beyond the scope of the current study, which focuses on early recruitment dynamics and cellular behavior at the injury site. Instead, we performed high-frequency live imaging (every 20 seconds for 1 hour) beginning at 1 hpi, enabling manual tracking of all neutrophils and macrophages entering the cornea during this window. While this represents a snapshot rather than a full resolution phase, it provides detailed insight into early post-injury immune cell behavior.

We have added the following in the discussion section:

Lines 509-515:

While our approach, which imaged immune dynamics only during the early post-injury window, does not allow definitive conclusions regarding neutrophil fate during inflammation resolution (e.g., reverse migration, apoptosis, or dispersal), it nevertheless captures the acute inflammatory response. Future studies employing photoconvertible reporters (such as Dendra2) and longer-term imaging will be well suited to address these questions and further refine the mechanisms of inflammatory resolution in the cornea (65).

6. Finally, the manuscript would benefit from a more explicit comparison of this MK model to mammalian systems and models, particularly regarding immune-cell kinetics and the overall inflammatory trajectory.

Thank you for the suggestion to compare our MK model's immune kinetics with mammalian systems. The inflammatory trajectory in our zebrafish model closely

parallels the kinetics of mammalian systems, where neutrophils serve as the primary responders. In mouse models, neutrophils are recruited as early as 2 hours post-abrasion *(Li et al., 2006)* or 6 hours following stromal LPS injection *(Carlson et al., 2006)*. While mammalian neutrophils typically peak at 24 hours and macrophages continue to rise over 7 days *(Chinnery et al., 2012),* our larval model exhibits a similar sequential pattern on an accelerated timescale. We have added the following to the discussion section:

Lines 517-526:

The immune-cell kinetics observed in our zebrafish larvae model closely recapitulate the fundamental inflammatory trajectory of mammalian systems, albeit on an accelerated timescale. In mouse models, neutrophils act as the primary responders, with infiltration occurring as early as 2 hours post-abrasion (38) or 6 hours following stromal LPS injection (66). We observe a similarly rapid primary neutrophil influx in the zebrafish, followed by a secondary wave of macrophages. While mammalian macrophage populations typically peak much later and continue to rise for up to 7 days post-insult (67), the zebrafish model demonstrates a compressed version of this hierarchical sequence. This conservation of temporal hierarchy underscores the translational utility of the zebrafish for modelling MK.

We sincerely thank the editor and reviewers for their time and careful, constructive evaluation of our manuscript. We greatly value their supportive and positive feedback, their recognition of the significance and utility of this model, and their insightful comments, which have greatly enhanced the clarity, flow, and overall quality of the paper.

Yours sincerely,

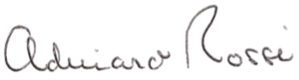

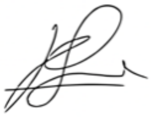

Professor Adriano G Rossi
Professor of Respiratory and Inflammation Pharmacology
Deputy Director of the Centre for Inflammation Research

Dr Kelvin Cheng
Wellcome Trust
Clinical Research Fellow

The University of Edinburgh is a charitable body, registered in Scotland, with registration number SC005336
